# Cellular Senescence Genes as Cutting‐Edge Signatures for Abdominal Aortic Aneurysm Diagnosis: Potential for Innovative Therapeutic Interventions

**DOI:** 10.1111/jcmm.70323

**Published:** 2025-01-17

**Authors:** Shuli Zhang, Jiayin Li, Ruichen Wang, Xiaojie Zhao, Zhu Mei, Xiaozeng Wang

**Affiliations:** ^1^ College of Medical and Bioinformatics Engineering Northeastern University Shenyang China; ^2^ State Key Laboratory of Frigid Zone Cardiovascular Diseases, Cardiovascular Research Institute and Department of Cardiology General Hospital of Northern Theater Command Shenyang China; ^3^ Northeastern University Shenyang China

**Keywords:** abdominal aortic aneurysm, biomarkers, cellular senescence‐related genes, ETS1, machine learning, therapeutic interventions

## Abstract

Abdominal aortic aneurysm (AAA) is the most prevalent dilated arterial aneurysm that poses a significant threat to older adults, but the molecular mechanisms linking senescence to AAA progression remain poorly understood. This study aims to identify cellular senescence‐related genes (SRGs) implicated in AAA development and assess their potential as therapeutic targets. Four hundred and twenty‐nine differentially expressed genes (DEGs) were identified from the GSE57691 training set, and 867 SRGs were obtained. Through the intersection of DEGs with SRGs, 19 differentially expressed senescence‐related genes (DESRGs) were uncovered. Functional enrichment analysis was performed to explore their biological roles in AAA. To identify hub genes, we applied machine learning algorithms, including LASSO, SVM‐RFE and random forest. These hub genes were then validated in two independent datasets. In the initial validation cohort, significant differences in the expression levels of BTG2, ETS1, ID1 and ITPR3 were observed between the AAA and control groups. Receiver operating characteristic (ROC) analysis demonstrated a robust diagnostic performance. Further validation across different AAA stages (small, large and ruptured AAA) identified ETS1 and ITPR3 as potential diagnostic genes. Subsequently, the diagnostic relevance of ETS1 and ITPR3 was further validated in human serum samples and mouse models of AAA. In addition, single‐cell RNA sequencing suggests that senescent endothelial cells play a pivotal role in AAA progression, we further confirmed the correlation between ETS1 and ITPR3 and senescent endothelial cells by WB, IF and RT‐qPCR. In conclusion, our study reveals the pivotal role of cellular senescence in AAA progression and identifies ETS1 and ITPR3 as promising diagnostic biomarkers.

**Abbreviations**
AAA: abdominal aortic aneurysm
AUC: area under the curve
BPs: biological processes
DEGs: differentially expressed genes
DESRGs: differentially expressed senescence‐related genes
ELISA: enzyme‐linked immunosorbent assay
ER: endoplasmic reticulum
GEO: Gene Expression Omnibus
GO: Gene Ontology
GSEA: gene set enrichment analysis
IF: immunofluorescence staining
IHC: immunohistochemistry
IP3R3: type 3 inositol 1,4,5‐trisphosphate receptor
KEGG: Kyoto Encyclopedia of Genes and Genomes
LASSO: least absolute shrinkage and selection operator
NO: nitric oxide
PCA: principal component analysis
PPI: protein‐–protein interaction
qPCR: quantitative polymerase chain reaction
RF: random forest
ROC: receiver operating characteristic
SASP: senescent‐associated secretory phenotype
scRNA‐seq: single‐cell RNA sequencing
SRGs: senescence‐related genes
SVM‐RFE: support vector machine‐recursive feature elimination
VSMCs: vascular smooth muscle cells
WB: western blotting

## 1 Introduction

Abdominal aortic aneurysm (AAA) is characterised by a focal expansion of the abdominal aorta, exceeding 50% of the normal arterial diameter [1]. The progression of AAA is usually insidious and asymptomatic, making early detection challenging. Imaging techniques are generally used to detect and monitor aneurysm size and progression, but they are often limited in their ability to predict the risk of rupture or identify AAA at an early stage before significant dilation occurs. These methods focus primarily on the anatomic features of aneurysms and may miss subtle or asymptomatic changes in the vasculature that occur prior to significant dilation. Moreover, these techniques are often costly, not universally accessible, and may expose patients to radiation or contrast agents, leading to potential complications [2, 3]. For patients with AAA < 5.5 cm, there are currently no accurate nonimaging methods for diagnosis, and clinical examinations are often unreliable [4, 5], leading to delays in necessary interventions. In contrast, for patients with AAA ≥ 5.5 cm and with low to moderate surgical risk, elective repair is recommended [6]. The risk of aneurysm rupture increases with diameter, with mortality rate reaching 90% following rupture [1]. The identification of specific biomarkers could offer a noninvasive and cost‐effective approach for early AAA detection, facilitating timely intervention and potentially improving patient outcomes. However, no biomarkers have been definitively established for the detection, progression or rupture of AAA, which significantly impairs timely diagnosis and rapid intervention [7]. Therefore, identifying biomarkers closely associated with the occurrence and rupture of AAA is essential to enhance detection accuracy and enable early warning and prediction [4].

Ageing is an independent risk factor for AAA occurrence [8, 9]. The incidence of AAA gradually increases with age. The prevalence of AAA in men aged > 65 years is up to 8%, with the incidence rate increasing by 40% every 5 years [10]. Ageing induces vascular senescence, leading to structural changes in the arterial, including an increase in collagen fibres and a decrease in the number of elastic fibres and smooth muscle cells [11]. Increased calcification of the vascular wall is another hallmark of vascular ageing [12]. In ageing arteries, there is a notable accumulation of senescent endothelial cells and vascular smooth muscle cells (VSMCs), characterised by a senescent‐associated secretory phenotype (SASP), manifesting as increased levels of reactive oxygen species, inflammation, cyclin‐dependent kinase inhibitors p16^INK4a^ and p21^CIP1^, phosphorylated p38, double‐stranded DNA breaks and increased SA‐β‐Gal activity [13, 14, 15]. Specifically, individuals diagnosed with AAA exhibit significantly shorter telomere length and higher oxidative DNA damage within their endothelial cells and VSMCs compared to healthy older individuals [16]. Recent studies have established a compelling association between cellular senescence and AAA pathogenesis. The accumulation of senescent vascular mesenchymal stromal cells over time impairs their remodelling ability during ageing, thereby promoting AAA initiation and progression [17]. Furthermore, the accumulation of senescent cells within the vascular wall and adjacent perivascular adipose tissue contributes to the development of aneurysms by triggering inflammation, oxidative stress and increasing leukocyte adhesion [18]. Therefore, exploring the molecular pathological changes in ageing vascular endothelial cells or VSMCs in AAA may provide important evidence for the identification of early biomarkers of AAA occurrence and rupture.

In this study, we initially employed bioinformatics analysis to process large‐scale gene expression datasets, which allowed us to efficiently screen for senescence‐related genes that might have a significant impact on AAA. Subsequently, we used three different machine learning algorithms to identify potential risk genes. The use of multiple algorithms allowed us to refine gene identification accuracy, ensuring the selection of the most relevant risk genes for AAA. Further, using two different validation sets, one comprising control and AAA samples, and the other comprising small, large and ruptured AAA, we identified ETS1 and ITPR3 as potential diagnostic genes for the occurrence and progression of AAA. We further validated the roles of ETS1 and ITPR3 in AAA through molecular experiments in human serum and mouse models of AAA. Next, to further investigate the functional roles of ETS1 and ITPR3, we conducted single‐cell RNA sequencing (scRNA‐seq) data analysis and built interaction networks of transcription factors (TF), gene and miRNA. This scRNA‐seq analysis indicates that senescent endothelial cells play a pivotal role in AAA progression. Finally, we validated the correlation between ETS1 and ITPR3 and senescent endothelial cells through western blot (WB), immunofluorescence (IF) and quantitative real time PCR (RT‐qPCR). These experiments validated the roles of ETS1 and ITPR3 at the protein and transcript level, solidifying their diagnostic and mechanistic relevance.

## 2 Materials and Methods

### 2.1 Dataset Acquisition and Processing

**FIGURE 1:**
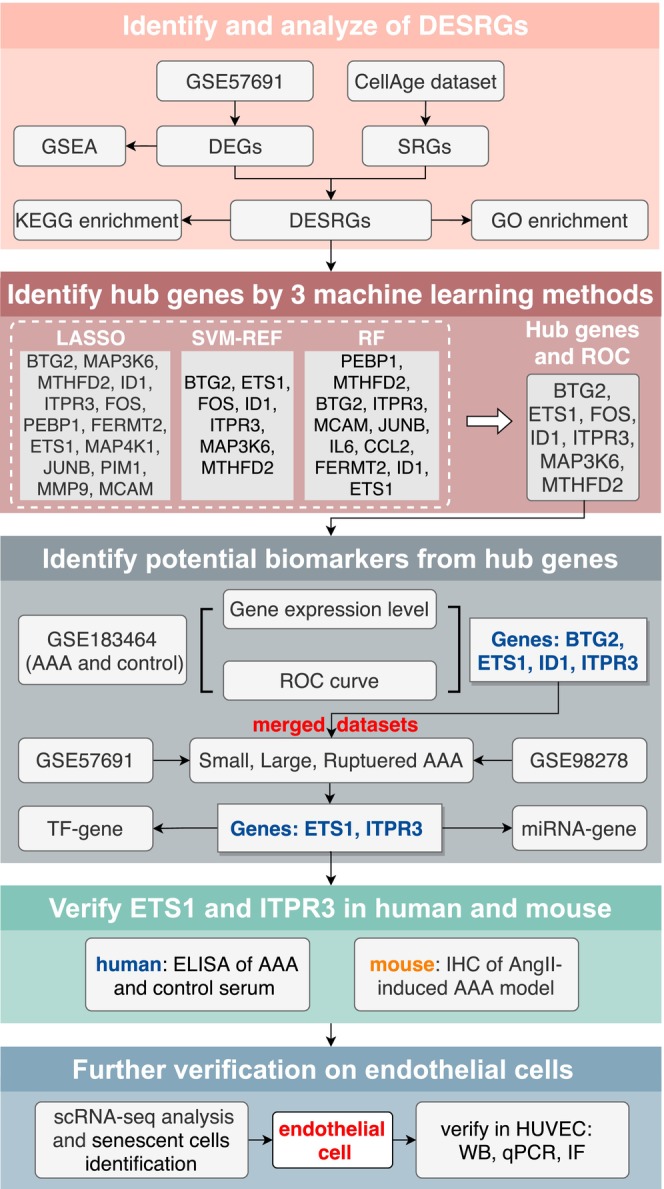
The flow chart of this study. AAA: Abdominal aortic aneurysm; BP, biological process; CC, cellular component; DEGs, differentially expressed genes; DESRGs, differentially expressed senescence‐related genes; GO, Gene Ontology; KEGG, Kyoto Encyclopedia of Genes and Genomes; MF, molecular function; SRGs, senescence‐related genes.

A comprehensive collection of 867 SRGs was obtained from the database of cell senescence genes (Table S3) [19]. Two microarray datasets, GSE57691 [20] and GSE98278 [21], as well as an RNA‐seq data, GSE183464 [22] were retrieved from the GEO database. The GSE57691 dataset includes 20 small AAA, 29 large AAA and 10 control samples. It served as both a training set and a validation set based on different classifications. The GSE183464 dataset was used to elucidate and validate the differences between AAA and control. We combine GSE98278 (including seven AAA and seven control samples) and GSE57691 (including 15 small AAA, 16 large AAA and 17 ruptured AAA) as the validation set. Then, the validation set was utilised to evaluate and validate the differences among small, large and ruptured AAA samples. The characteristics of these datasets that we have described can also be seen in Table S1. Each gene in these datasets was annotated using their respective platforms. The batch effect between GSE57691 and GSE98278 was removed using the function ‘removeBatchEffect’ in limma (3.56.2) package [23]. Subsequently, the data were merged, and 35 small AAA, 45 large AAA and 16 ruptured AAA samples were obtained as merged datasets to identify the different degrees of AAA gene modules. Next, the limma package in R (4.3.1) was used for quality control and normalisation of the gene expression profiles, which are represented by boxplots. Principal component analysis (PCA) was performed using ‘FactoMineR (2.9)’ to verify the reproducibility of the data, and the PCA plot was constructed using the ‘factoextra (1.0.7)’ R package. Figure 1 illustrates the flow chart of this study.

### 2.2 Analysis of Differentially Expressed Genes (DEGs)

The limma R package was used to investigate the differentially expressed genes (DEGs) between the AAA and control samples in the training set of GSE57691. Volcano and difference ranking plots were generated by ‘ggplot2 (3.4.4)’ whereas heatmap plots were created using the ‘Heatmap (1.0.12)’. DEGs were identified based on the criteria |log_2_(FC)| > 1 and *p*‐value < 0.05. Gene set enrichment analysis (GSEA) was performed using the ‘clusterProfiler (4.8.3)’ package [24], and the enriched pathways of GSEA were screened and identified using a false discovery rate < 0.25 and a *p*‐value < 0.05.

### 2.3 Analysis of the Differential Expression of Senescence‐Related Genes (DESREGs)

The ‘venn (1.11)’ and ‘VennDiagram (1.7.3)’ packages in R were used to draw the intersection of DEGs and SRGs, resulting in the identification of differentially expressed SRGs (DESRGs). The protein–protein interaction (PPI) network of the DESRGs was constructed using the STRING database (https://string‐db.org/). Spearman's correlations among the DESRGs were calculated. Further analysis of the DESRGs involved the Kyoto Encyclopedia of Genes and Genomes (KEGG) pathway and gene ontology (GO) enrichment analyses, which were performed using ‘clusterProfiler’ and ‘GOplot (1.0.2)’.

### 2.4 Identification of Hub Genes Combining Three Machine Learning Techniques

Three machine learning algorithms, including least absolute shrinkage and selection operator (LASSO), support vector machine‐recursive feature elimination (SVM‐RFE) and random forest (RF), were used to investigate disease status. The LASSO algorithm was utilised for variable selection and complexity adjustment [25]. It is a variant of linear regression that introduces an L1 regularisation term into the loss function to achieve feature sparsity, effectively setting the weights of some features to zero, thus performing feature selection. The SVM‐RFE algorithm was used to identify the most relevant key genes due to its robust feature selection capabilities, especially in classification tasks [26]. In our study, we carried out feature extraction of candidate genes by this method. The RF algorithm aims to derive the most reliable outcomes by leveraging numerous underlying tree models [27]. RF demonstrates strong versatility and robustness in both classification and regression tasks. We have used 10‐fold cross‐validation and independent validation sets to verify the stability and predictive capabilities of the models. The results of the 10‐fold cross‐validation showed that the accuracy and Kappa scores for each algorithm were above 0.9 (Figure S4A), where the Kappa coefficient is a measure of classification accuracy. Similarly, the evaluation on the independent test set demonstrated consistent accuracy exceeding 0.9 across all three algorithms (Figure S4B). Our comprehensive comparison and evaluation demonstrate that all models have high accuracy and kappa values. This confirms the robustness and high predictive accuracy of the LASSO, random forest and SVM‐REF models for identifying hub genes to diagnose AAA. Finally, the optimal hub genes were determined by identifying the common genes resulting from the intersection of these algorithms.

### 2.5 Identification and Verification of Diagnostic Genes From Hub Genes

To identify diagnostic genes, scatterplots and boxplots were used to visually display the expression levels of hub genes in patients with varying degrees of AAA. Receiver operating characteristic (ROC) curve analysis was performed, and the areas under the curve (AUCs) were calculated using the ‘pROC (1.18.5)’, which is credible to determine the predictive capability of the hub genes [28]. The area AUC under the ROC curve is between 0.5 and 1, which can be used as a value to intuitively evaluate the classification. The larger the value, the better it is, and the higher the accuracy (above 0.6 is preferred) [29]. Diagnostic genes were selected from the training and validation sets according to the AUC criterion. To investigate the dynamic changes in diagnostic genes associated with AAA, the expression levels and ROC curves of these genes were analysed to different degrees in humans with AAA and at different time points in mice with AAA.

### 2.6 Single‐Cell RNA Sequencing Data Analysis and Identification of Senescent Cell

To further explore and validate the distribution of ETS1 and ITPR3 in AAA, the single‐cell RNA sequencing (scRNA‐seq) dataset GSE166676 [30] was obtained from the GEO database for analysis. The scRNA‐seq data were converted into Seurat objects through ‘CreateSeuratObject’ function in the Seurat package (version 4.0.0) [31]. Cells with < 200 or > 2500 genes detected, and mitochondrial gene > 25% were considered to be low‐quality cells and filtered out, resulting in 11,785 cells. Subsequently, the data were subjected to dimension reduction, including variable gene selection, principal component analysis (PCA) and uniform manifold approximation and projection (UMAP) [32]. Subsequently, we evaluated cell clustering across a range of predetermined resolution scales to ensure the separation of the well‐known major aortic cell types. Cell types were identified based on their expression levels of cell‐specific markers, as previously reported in the literature. Then, senescent cells were identified by senescent cell identification (SenCID) [33].

### 2.7 Construction of the TF‐Gene, miRNA‐Gene and Gene–Gene Interaction Network

The regulatory associations between the diagnostic genes and TFs were obtained from the JASPAR database [34]. Additionally, the TarBase [35] database was used to construct a gene–miRNA interactions network to predict the correlation between diagnostic genes and miRNAs [36]. These networks were constructed in their minimum forms by the NetworkAnalyst 3.0 [37], and visualised by Cytoscape. GeneMANIA [38] (http://www.genemania.org) was used to construct the gene–gene interaction network.

### 2.8 Enzyme‐Linked Immunosorbent Assay (ELISA)

In total, 29 patients with AAA (median age, 70 years; 100% male) and 29 controls (the diameter of the abdominal aorta is not dilated and the abdominal aortic wall is structured; median age, 64 years; 100% male) were enrolled. All serum samples were collected from the General Hospital of the Northern Theatre of Operations for enzyme‐linked immunosorbent analysis (ELISA). Serum ETS1 and ITPR3 concentrations were quantified using a commercial human ETS1 ELISA kit (LV11431, Animalunion Biotechnology) and a human ITPR3 ELISA kit (LV11432, Animalunion Biotechnology), respectively, following the manufacturer's instructions.

### 2.9 Experimental Animals and Models

The male Apolipoprotein E knockout (ApoE^−/−^) mice used in this study were purchased from GemPharmatech. All mice were housed in a pathogen‐free environment with a temperature of 22°C–24°C and a humidity of 60%–65%, fed normally and the experiment began after 1 week of growth. All experiments were approved by the Ethics Committee of the Northern Theatre General Hospital and were conducted in accordance with current guidelines on the care and use of laboratory animals. All animal care and testing protocols adhere to the National Institutes of Health Guidelines for the Care and use of laboratory animals.

Angiotensin II (Ang II) (1000 ng/kg/min; A1042, APExBIO Technology) was dissolved in normal saline and infused with an osmotic pump (Alzet model 1004; AlzaCorp) in 8‐week‐old ApoE^−/−^ mice for 28 days. The aortic diameter was measured using the small animal ultrasound system Vevo 2100 (Visual Sonics) by a blind researcher. Further, aortic tissues were harvested for histological analyses.

### 2.10 Cell Culture and Treatment

Human umbilical vein endothelial cells (HUVEC) were cultured in endothelial cell medium (1001, ScienCell). We used HUVEC between the sixth and eighth passages for experiments. The cells were seeded into six‐well plates and treated with H_2_O_2_ (660 μM) for 12 h. The cell medium was changed and treated with H_2_O_2_ (660 μM) for 12 h again.

### 2.11 Immunofluorescence Staining (IF)

Germ‐free coverslips were placed in six‐well plates, and the cells were cultured as mentioned above. After culturing, the cells were fixed in 4% paraformaldehyde for 30 min at room temperature. Subsequently, the cells were washed 3 times in PBS and incubated with 0.1% Triton X‐100 for 15 min, then were incubated with rabbit anti‐ETS antibody (1:100; A19603, ABclonal), or rabbit anti‐ITPR3 antibody (1:100; A23202, ABclonal) overnight and then incubated with goat antirabbit secondary antibody for 1 h at room temperature. Last, the cells were incubated with DAPI for 5 min. After staining, the coverslips were carefully removed and the cells were mounted using ProLong Gold antifade reagent (Thermo Fisher Scientific). Finally, samples were imaged using a fluorescence microscope (Zeiss).

### 2.12 Immunohistochemistry (IHC)

Paraffin sections of the mouse aorta were dehydrated. Antigen repair was performed and endogenous peroxidase was added (immunohistochemistry kit, MXB Biotechnologies). Nonspecific staining was blocked with nonimmune goat serum. The sections were incubated at room temperature for 1 h with rabbit anti‐ETS antibody (1:100; A19603, ABclonal), or rabbit anti‐ITPR3 antibody (1:100; A23202, ABclonal). Sheep antimouse/rabbit IgG polymer labelled with biotin is extracted at room temperature for 1 h. All specimens were reversed stained with DAB and haematoxylin, dehydrated, mounted on slides and then photographed using a microscope (Zeiss). Two independent, blinded researchers randomly evaluated areas on at least three slides.

### 2.13 RNA Isolation and Quantitative Polymerase Chain Reaction (qPCR)

Total RNA was extracted from the collected cells using TRIzol reagent (Invitrogen), following the manufacturer's protocol. The extracted RNA was then reverse‐transcribed into complementary DNA (cDNA) utilising the PrimeScript RT reagent Kit (Takara, Beijing, China). Quantitative real‐time PCR (qRT‐PCR) was performed on a CFX96 Touch Real‐Time PCR Detection System (Bio‐Rad, Hercules, CA, USA). The reaction mixture consisted of TB Green Fast qPCR Mix (Takara) and specific primers designed by Ribobio. The primer sequences used as follows: ITPR3 (forward 5′‐AGTTCCTGACGTGTGACGAG‐3′; reverse 5′‐TGAAGCGGTACAAGCCATTCC‐3′) and ETS1 (forward 5′‐GATAGTTGTGATCGCCTCACC ‐3′; reverse 5′‐GTCCTCTGAGTCGAAGCTGTC ‐3′).

### 2.14 Western Blot Analysis

Cells were homogenised in RIPA buffer (Thermo Fisher Scientific, Glen Burnie, MD, USA) supplemented with protease and phosphatase inhibitors. Protein concentrations were determined using the BCA Protein Assay Reagent Kit (Thermo Fisher Scientific). For protein expression analysis, 15 μg of total protein was used. Specific antibodies against ETS1 (A19603, ABclonal), ITPR3 (A23202, ABclonal), GAPDH (SA30‐01, Thermo Fisher Scientific), Tubulin (catalogue number not provided) and P21 (sc‐471, Santa Cruz Biotechnology) were employed.

### 2.15 Statistical Analyses

R 4.3.1 software and Microsoft PowerPoint 2022 were used in this study. Data are presented as the means ± standard deviations, and statistical comparisons between groups were performed using an unpaired Student's *t*‐test. A *p*‐value of < 0.05 was considered statistically significant. ROCs were used to evaluate the AUCs and predictive abilities. A linear regression model was used to analyse the correlation between the expression of EST1 and ITPR3 in the serum of AAA patients and patients' age, length of AAA and CT‐measured vessel diameter. The *p*‐value was calculated by Pearson correlation.

## 3 Results

### 3.1 Identification of DEGs and DESRGs in the AAA and Normal Samples

**FIGURE 2:**
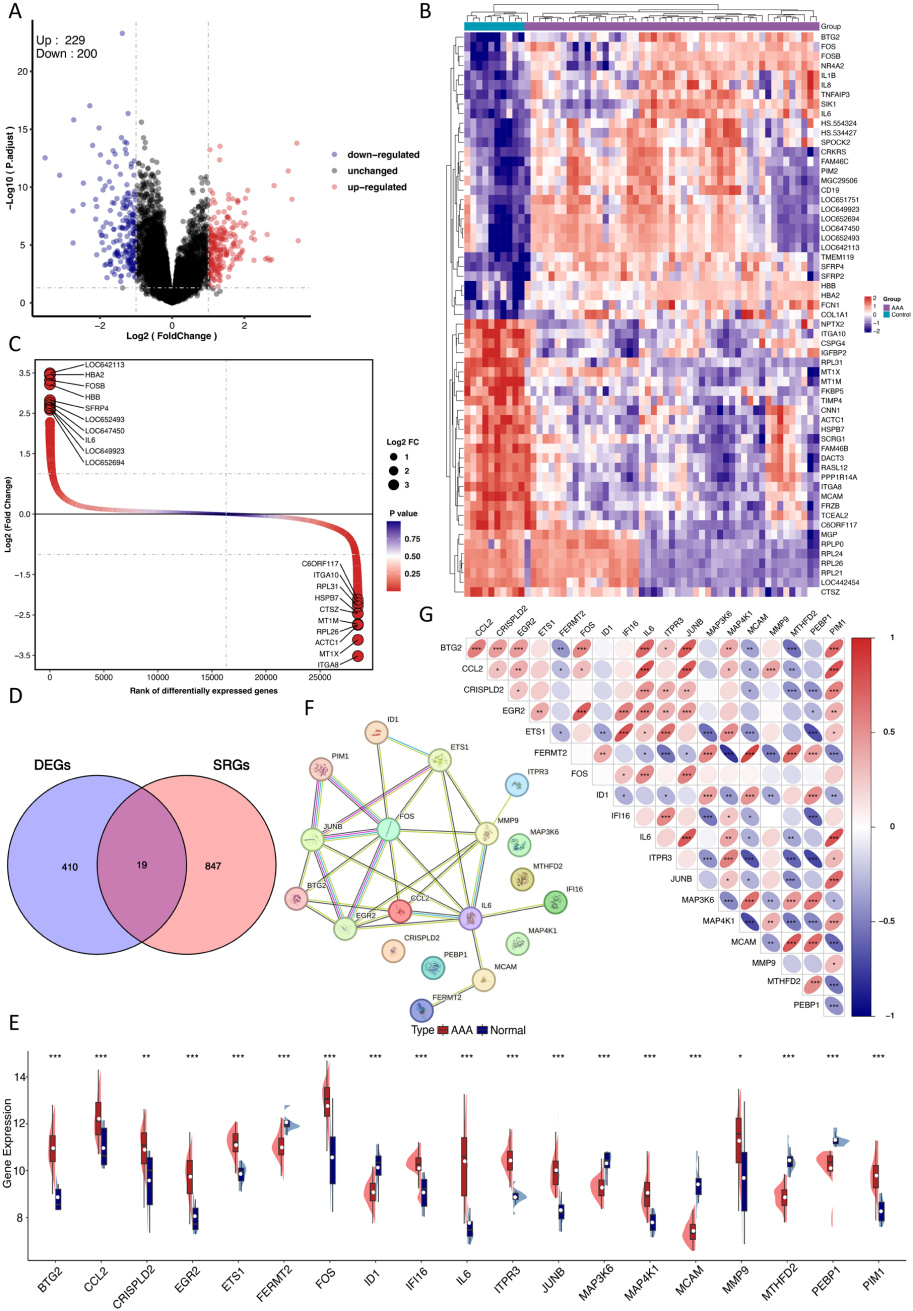
Identification of DEGs and DESRGs in the GSE57691 training set and the expression and correlation analysis of 19 DESRGs. (A) Volcano plot of DEGs between the AAA and control samples. (B) Heatmap of top 60 DEGs. (C) Difference ranking plot of top 10 DEGs. (D) Venn diagram of overlapping genes between the GSE57691 training set and CellAge database. (E) Expression levels of DESRGs in the AAA and control samples in the GSE57691 training set were visualised by box plot. (F) Protein–protein interaction (PPI) network constituted with the DESRGs. (G) Spearman correlation among 19 DESRGs. AAA, abdominal aortic aneurysm; DESRGs, differentially expressed senescence‐related genes. DEGs, differentially expressed genes; SRGs, senescence‐related genes. *p < 0.05, **p < 0.01, ***p < 0.001.

To identify potential candidate genes from complex high‐throughput sequencing data, we initially conducted a comprehensive bioinformatics analysis. Normalisation was performed on the expression matrices of the GSE57691, GSE183464 and GSE98278 datasets. The boxplots exhibited a distribution trend characterised by large straight lines (Figure S1A–C). PCA demonstrated that the samples were different between groups and very similar within groups (Figure S1D–F). The boxplot and PCA of our merged datasets are shown in Figure S2. By applying the thresholds of |log_2_(FC)| > 1 and *p*‐value < 0.05, 429 DEGs were identified from the GSE57691 training set. Among these DEGs, 229 genes were significantly upregulated, while 200 genes were significantly downregulated (Table S4), the symbols, logFC, AveExpr, *p*‐value and adj‐*p*‐value of these DEGs are recorded in Table S4. Volcano plots and heatmaps were used to visualise DEGs (Figure 2A,B), and the difference ranking plot highlighted the top 10 upregulated and downregulated genes (Figure 2C). GSEA revealed that the DEGs were primarily associated with the regulation of immune system processes, innate immune system, immune system and cytokine signalling in the immune system (Figure S3A–D). Next, 19 overlapping DESRGs were identified by combining the DEGs with the SRGs for further investigation (Figure 2D). Six DESRGs were downregulated, and 19 DESRGs were upregulated (Table S2). The expression levels and correlations of the 19 DESRGs in the GSE57691 training set are shown in Figure 2E,G. There is high correlation between BTG2 and CCL2, which is one of the most well‐known CC chemokines. Additionally, FOS was strongly associated with IL6, a key component of the SASP. The STRING database was utilised to construct the PPI network, facilitating the identification of interactive relationships among DESRGs. The constructed PPI network consisted of 19 nodes and 29 edges (Figure 2F). Table S2 provides detailed information on the 19 DESRGs, including their roles in senescence and their expression changes observed in the GSE57691 training set.

### 3.2 Functional Enrichment Analysis of 19 DESRGs

**FIGURE 3:**
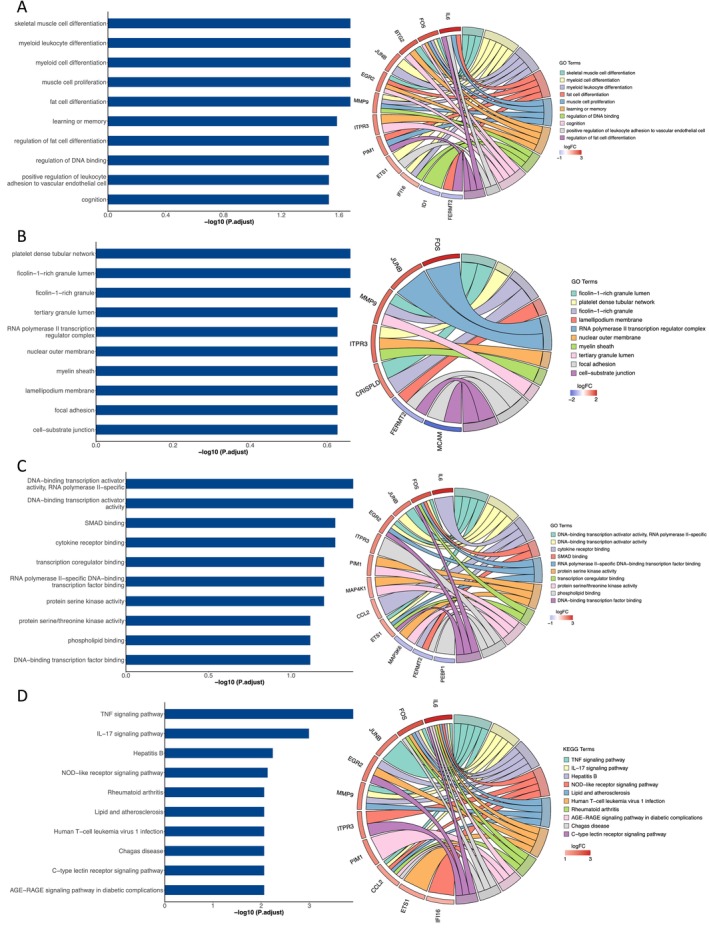
Bar plots and Chord plots of 19 DESRGs enriched GO terms and KEGG pathways. (A–D) represents BP, CC, MF and KEGG respectively. BP, biological process; CC, cellular component; DESRGs, differentially expressed senescence‐related genes; GO, Gene Ontology; KEGG, Kyoto Encyclopedia of Genes and Genomes; MF, molecular function.

Functional annotation and pathway analysis uncover the underlying biological mechanisms. Therefore, we further performed GO and KEGG analyses for DESRGs (Figure 3). The biological processes (BPs) of DESRGs were primarily associated with various cell differentiations, including skeletal muscle cells, myeloid leukocyte, myeloid cells, muscle cells and fat cells. Regarding cellular component, DESRGs were predominantly enriched in platelet dense tubular network, ficolin‐1‐rich granule lumen, tertiary granule lumen, RNA polymerase II transcription and regulator complex. The DESRGs were enriched in various molecular functions, such as SMAD binding, cytokine receptor binding, transcription coregulator binding, protein serine/threonine kinase activity and phospholipid binding. KEGG analysis revealed that the DESRGs were predominantly associated with the TNF and NOD‐like receptor signalling pathways. The TNF signalling pathway primarily regulates immune cell functions, while NOD‐like receptors serve as crucial components of the innate immune system in mammals, governing immune and inflammatory responses. The research has already linked vascular ageing to inflammation [39], and our results confirm this. In addition, lipid and atherosclerosis, C‐type lectin receptor signalling pathway and AGE‐RAGE signalling pathway in diabetic complications were also enriched in KEGG.

### 3.3 Identification and Evaluation of Seven Common Hub Genes From 19 DESRGs by ML

Machine learning is uniquely capable of detecting patterns in data and making predictions, enabling the identification of the most functional and diagnostically relevant genes from a set of candidate genes. To ensure robust and accurate identification, we employed a combination of feature selection methods, including SVM‐RFE, LASSO and RF. These complementary approaches allowed for the thorough assessment of relevant features, enhancing the reliability and validity of our findings.

**FIGURE 4:**
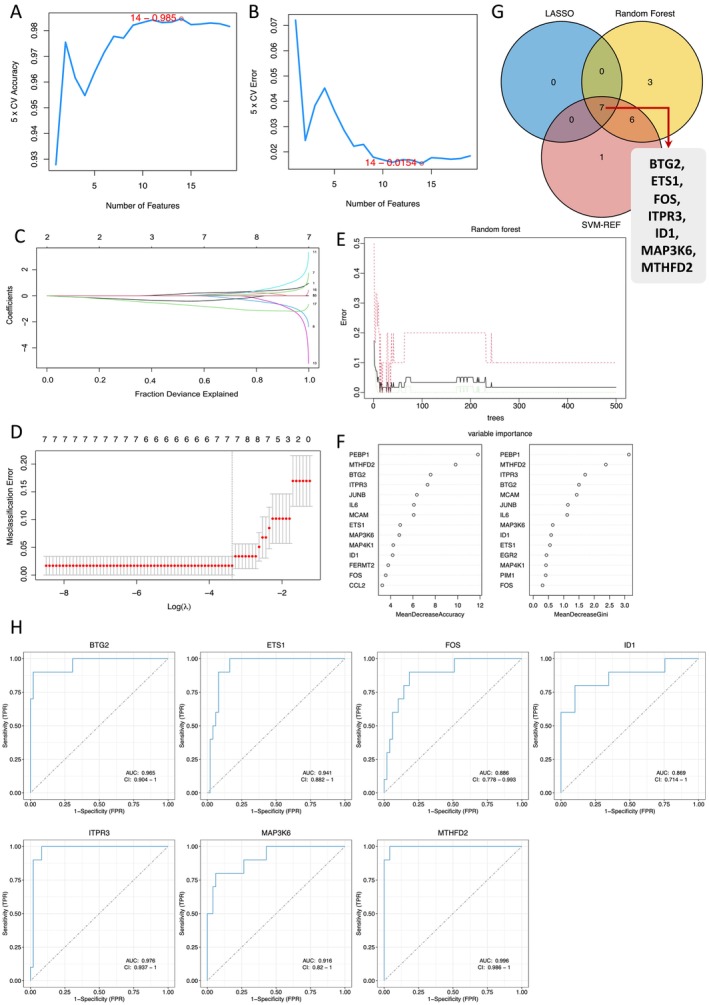
Three machine learning algorithms were applied to explore the optimal hub genes, further the diagnostic value of seven hub genes in the GSE57691 training set was evaluated. (A, B) The SVM‐RFE algorithm was performed to further candidate optimal feature genes with the highest accuracy and lowest error obtained in the curves. (C, D) The LASSO algorithm determined the candidate optimal feature genes and the optimal lambda. Each coefficient curve in the left picture represents a single gene. The dashed vertical lines represent the partial likelihood deviance. (E) Random forest for the relationships between the number of trees and error rate. (F) Top 10 key genes with MeanDecreaseAccuracy and MeanDecreaseGini were identified in the RF algorithm. (G) Venn diagram displays the seven optimal key genes overlapped by LASSO, random forest and SVM‐REF algorithms. (H) ROC curves of BTG2, ETS1, FOS, ID1, ITPR3, MAP3K6 and MTHFD2 in the GSE57691 training set. ROC, receiver operating characteristic.

SVM‐RFE recursively removes the least important features and builds models on the remaining features, which is effective in selecting a subset of relevant features. Based on the results of the SVM, as shown in the error rate plot (Figure 4B), the position of the red circle indicates the lowest error rate, which occurs when using 14 genes, with an error rate of 0.015. Therefore, we selected 14 genes as our screening criteria, achieving an accuracy of 0.985 (Figure 4A). For the LASSO algorithm, the dashed line in panel Figure 4D corresponds to the parameter ‘lambda.min’, which serves as a threshold determining how many gene coefficients can be retained. From Figure 4D, it can be seen that the value corresponding to the dashed line is 7, indicating that seven genes are retained as the final selected feature genes. This ensemble learning method provides importance scores for each feature, which helps in ranking and selecting the most important genes. As for the RF, Mean Decrease Accuracy and Mean Decrease Gini are two metrics indicating the importance of variables in the RF results. Both metrics suggest that the higher the value, the more important the variable. Therefore, we selected the top 10 genes based on both ‘Mean Decrease Accuracy’ and ‘Mean Decrease Gini’ scores (Figure 4F), and identified their intersection as the feature genes selected by the RF algorithm.

Subsequently, the key genes obtained from these machine learning algorithms were comprehensively compared, resulting in the identification of seven optimal key genes. These genes included BTG2, ETS1, FOS, ID1, ITPR3, MAP3K6 and MTHFD2 (Figure 4G). Given the complexity of AAA and the potential variability in datasets, there is a risk of overfitting or model generalisation issues. To mitigate this, ROC curves were used to evaluate the diagnostic value of seven hub genes for AAA. Figure 4H shows the diagnostic values of these genes in the GSE57691 set. The results showed that all hub genes had an AUC > 0.850, indicating their potential as reliable biomarkers.

### 3.4 Two Diagnostic Genes (ETS1 and ITPR3) Closely Associated With AAA Occurrence and Development

**FIGURE 5:**
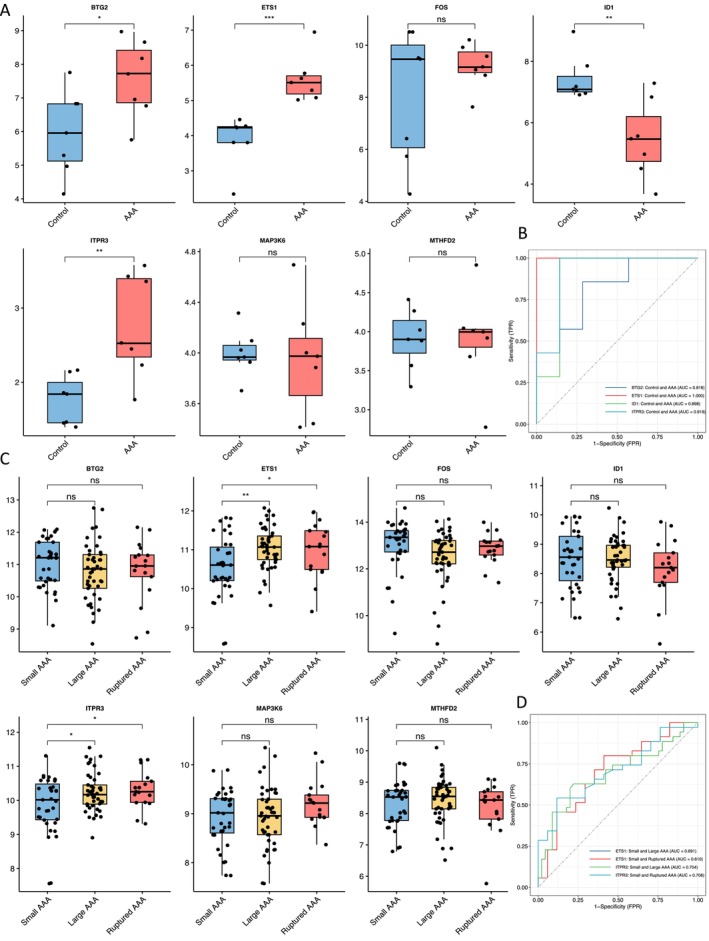
Comparison of the expressions and diagnostic value of seven hub genes in the first validation set GSE183464 and merged datasets of GSE57691 and GSE98278. (A) Expression levels of BTG2, ETS1, FOS, ID1, ITPR3, MAP3K6 and MTHFD2 in GSE183464. (B) The ROC curves of BTG2, ETS1, ID1 and ITPR3 in GSE183464. (C) Expression levels of BTG2, ETS1, FOS, ID1, ITPR3, MAP3K6 and MTHFD2 in merged datasets. (D) The ROC curves of ETS1 and ITPR3 in small AAA, large AAA and ruptured AAA of merged datasets. ROC, receiver operating characteristic. ns, no significance; *p < 0.05; **p < 0.01; ***p < 0.001.

It is important to further validate the seven hub genes across different datasets. The gene expression levels and diagnostic values were first verified in the validation set of GSE183464, which consists of seven AAA and seven normal samples. Significant differences were observed in the expression levels of BTG2, ETS1, ID1 and ITPR3 (Figure 5A). ETS1 showed the highest diagnostic value, with an AUC of 1.000. ITPR3 had the second highest AUC of 0.918 (Figure 5B). Furthermore, during the merged dataset validation analysis combining GSE57691 and GSE98278, we observed significant differences in the expression levels of ETS1 and ITPR3 when comparing small AAA with large AAA and small AAA with ruptured AAA (Figure 5C). Both genes had an AUC of > 0.600 (Figure 5D). The AUC of ETS1 was 0.691 and 0.610 in small AAA versus large AAA and small AAA versus ruptured AAA respectively. ITPR3 had greater diagnostic potential, with an AUC of 0.704 and 0.708 in small AAA versus large AAA and small AAA versus ruptured AAA. These findings, combined with the previously discussed results, underscore the significance of ETS1 and ITPR3 as the two selected diagnostic genes.

### 3.5 Construction of the TF‐Gene, miRNA‐Gene and Gene–Gene Networks of ETS1 and ITPR3

To explore the potential regulatory relationships involving the diagnostic genes, the JASPAR database was used to predict the TFs that interact with ETS1 and ITPR3. The results were subsequently visualised using Cytoscape, producing a network diagram consisting of 21 nodes and 23 edges (Figure S5B). Our analysis revealed that the transcription factors E2F1, GATA2, HINFP and POU2F2 were identified as common regulators of both diagnostic genes.

Furthermore, the TarBase database and CTD tools were used to predict the target miRNAs associated with the two diagnostic genes. In total, 101 miRNAs were identified (Figure S5C). Specifically, ITPR3 was regulated by 29 miRNAs, and ETS1 was targeted by 80 miRNAs. Several miRNAs, including hsa‐mir‐34a‐5p, hsa‐mir‐182‐5p, hsa‐mir‐129‐2‐3p, hsa‐mir‐1‐3p, hsa‐mir‐214‐3p, hsa‐mir‐374a‐5p, hsa‐let‐7d‐5p and hsa‐mir‐124‐3p, were found to be simultaneously regulated by both genes.

In addition to exploring the regulatory relationship among genes, TF and miRNA, we generated a gene–gene interaction network using GeneMANIA to uncover altered neighbouring genes associated with ETS1 and ITPR3 (Figure S5D,E). The results showed that the 20 most frequently and closely correlated genes with ETS1 included NFKB1, MAPK1, MAPK3 and GFI1. Functional analysis indicated significant associations between the positive regulation of cell migration, regulation of steroid biosynthetic processes and other biological functions. Similarly, for ITPR3, correlated genes, including ATP2A2, ITPR2 and ASPH, were implicated in functions related to calcium ion transmembrane transporter activity, cardiac conduction and others. These findings provide a comprehensive understanding of the intricate regulatory landscape surrounding ETS1 and ITPR3 in AAA.

### 3.6 Validation of Diagnostic Genes ETS1 and ITPR3

**FIGURE 6:**
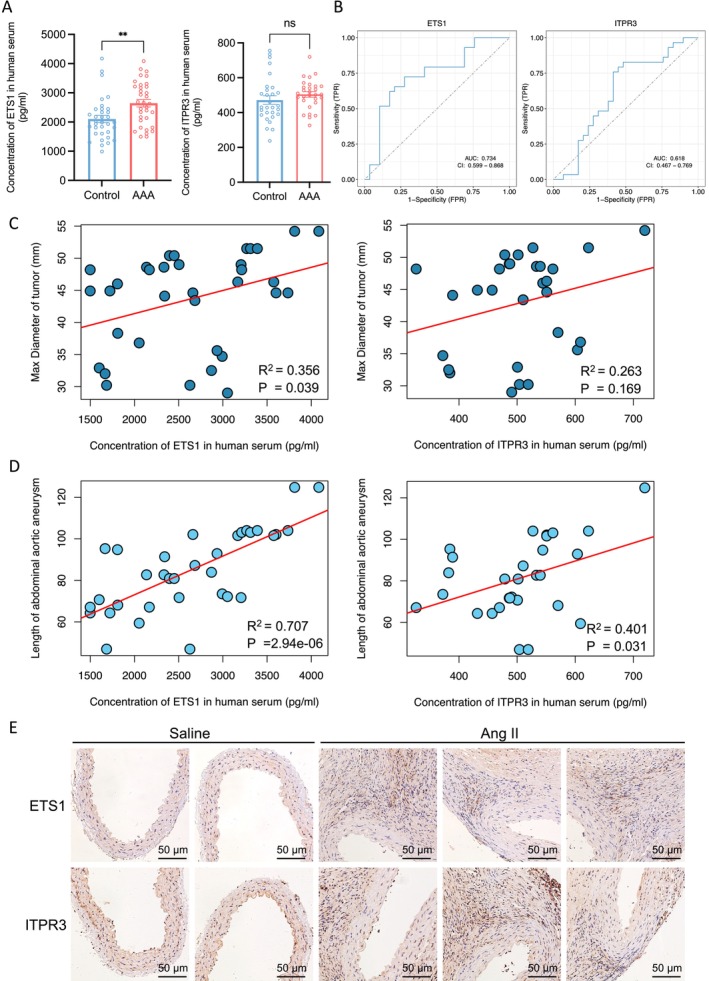
Validation of the ETS1 and ITPR3 in human and mouse. (A) Boxplot showing the protein level of ETS1 and ITPR3 in patients of AAA (*N* = 34) and control serum (*N* = 29). (B) The ROC curves of ETS1 and ITPR3 in human serum. (C) Correlation of concentration of ETS1 (left panel) and ITPR3 (right panel) in human serum (pg/mL) with max diameter of tumour (mm). (D) Correlation of concentration of ETS1 (left panel) and ITPR3 (right panel) in human serum (pg/mL) with length of AAA (mm). The *p*‐value was calculated by Pearson correlation. (E) IHC of ETS1 and ITPR3 in Ang‐II induced AAA and normal mouse aorta. ns, no significance; **p < 0.01.

Molecular biology experiments play a crucial role in validating the reliability and biological significance of ETS1 and ITPR3. To further validate the diagnostic effect in human AAA, we detected the protein expression levels of ETS1 and ITPR3 in the serum of 29 patients with AAA and 29 control participants. Our findings revealed a statistically significant upregulation of ETS1 expression in AAA patients compared to the control group (*p* = 0.0024). Additionally, ITPR3 also demonstrates an elevated expression in patients with AAA (Figure 6A). Further, we performed AUC analysis on human ELISA data to comprehensively evaluate the diagnostic performance of ETS1 and ITPR3. The results indicate that ETS1 demonstrates a higher diagnostic potential compared to ITPR3 (Figure 6B).

In parallel, we constructed AAA animal models to further investigate the functional roles of ETS1 and ITPR3. we performed immunohistochemistry on vascular tissues from AngII‐induced AAA mice and normal mice. We found that ETS1 and ITPR3 were significantly elevated in AAA vascular tissues (Figure 6C). These results consistently prove the correlation between ETS1 and ITPR3 and AAA.

### 3.7 Single‐Cell RNA Sequencing Demonstrating the Cellular Distribution of ETS1 and ITPR3

**FIGURE 7:**
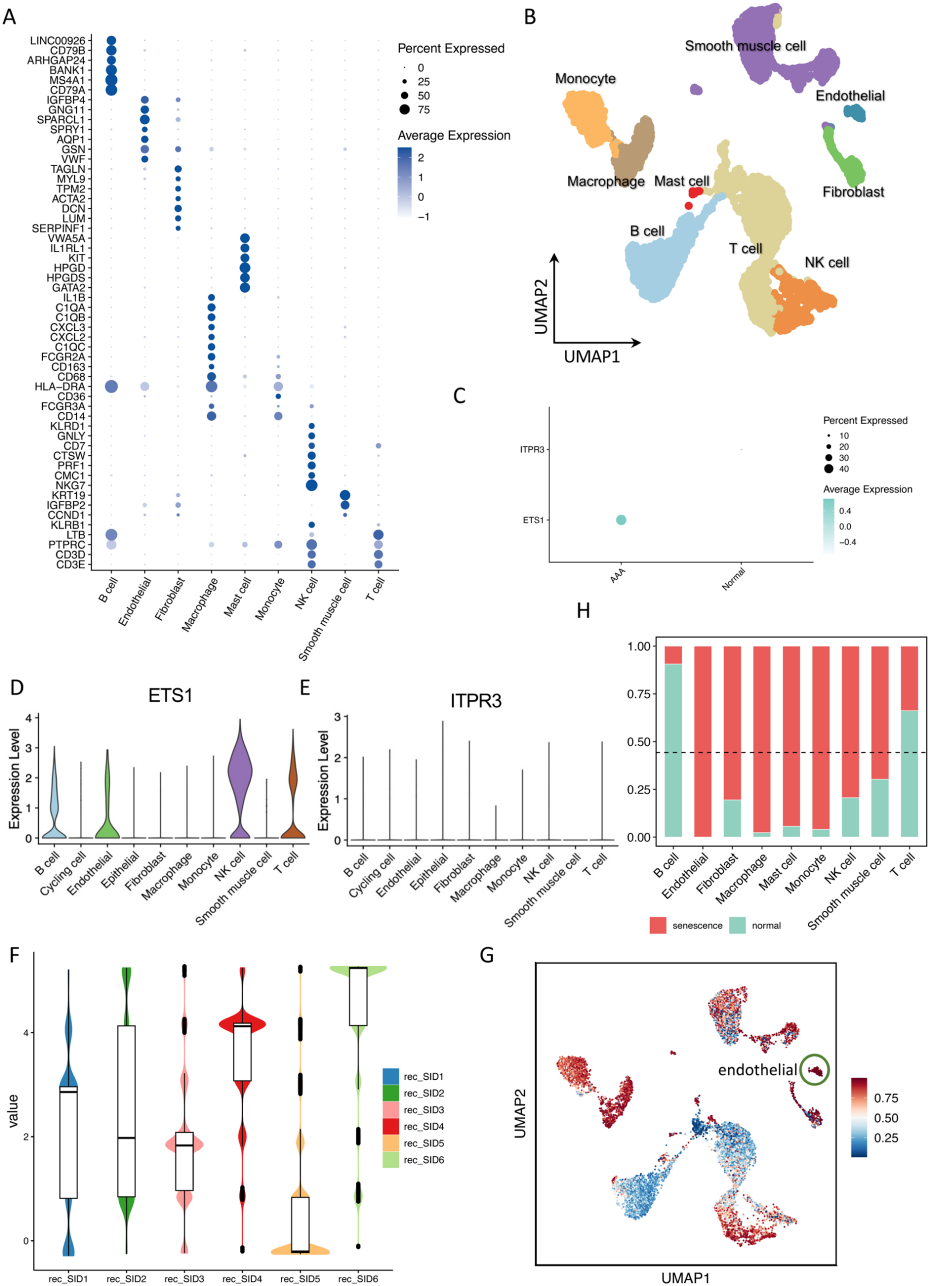
Identification of senescent cells from scRNA‐seq data. (A) Dot plot showing expression of marker genes in each cell type. (B) UMAP plot showing that aortic tissues were divided into nine distinct cell types. (C) The expression of ETS1 and ITPR3 in AAA and control. Gene expression levels of ETS1 (D) and ITPR3 (E) in different cell types are shown. (F) SID6 gets the highest recommendation score from SenCID. The value of the ordinate represents the recommendation score given by the SenCID. (G) UMAP showed ageing score of each cell from SID6 model. (H) The proportion of senescent cells in each cell type. The dashed line represents the total senescent cell proportion (0.443).

scRNA‐seq is a powerful approach for dissecting and comprehending cellular transcriptomic heterogeneity within tissues at the resolution of individual cells. To delineate the expression patterns of ETS1 and ITPR3 in distinct cell populations within aortic tissues from patients with AAA and healthy individuals, we used the scRNA‐seq dataset GSE166676, comprising samples from two healthy volunteers and four patients with AAA [30]. Different cell types were defined with precision and clarity using lineage‐specific biomarkers according to previous studies (Figure 7A,B) [40]. The analysis revealed a notable upregulation of ETS1 expression in AAA samples compared with that in controls, consistent with the findings from bulk RNA‐seq (Figure 7C). Furthermore, the spatial distribution of ETS1‐expressing cells exhibited a strong colocalisation with clusters identified as endothelial and immune cells (including T cells, B cells and NK cells), consistent with previous findings [41, 42] (Figure 6D,E). Next, we performed GO and KEGG enrichment analyses on the endothelial and NK cell subpopulations to elucidate pathways involving ETS1. The results revealed that ETS1 is involved in several critical pathways, including regulation of vasculature development, regulation of angiogenesis, blood vessel endothelial cell migration, cellular senescence and the Ras signalling pathway (Figure S6, Table S5). The enrichment of these pathways is recorded in detail in Table S5. These results highlights the potential involvement of specific immune cell subsets in driving the upregulation of ETS1 within the aortic microenvironment.

### 3.8 Senescent Endothelial Cells Play an Important Role in AAA

SenCID is a newly developed machine learning programme designed to precisely identify senescent cells in both somatic and single‐cell transcriptomes. It provides the first demonstration that cells can be categorised into six distinct SID classes [33]. SenCID results indicate that SID6 is deemed most suitable for senescence evaluation, receiving the highest recommendation score (Figure 7F). According to SenCID, there are six SID models available to characterise senescence across various cell types: SID1, SID2, SID3, SID4, SID5 and SID6. Each cell obtained an ageing score from 0 to 1, and the closer the value is to 1, the more likely it is an ageing cell. The ageing score of each cell based on the SID6 model was mapped onto UMAP (Figure 7G). Additionally, each cell is assigned a binary score, wherein 1 indicates senescent cells and 0 denotes normal cells. Statistical analysis revealed that out of 11,785 cells, 5223 cells were classified as senescent (44.3%). It is worth mentioning that all endothelial cells are considered senescent cells (Figure 7H), and previous studies have confirmed that aged arteries are characterised by the presence of accumulation of senescent endothelial and senescent vascular smooth cells [15, 39]. Furthermore, the proportion of senescent cells in macrophages and monocytes was notably high (Figure 7H). Research has indicated that the formation of AAA is a complex process involving the infiltration of inflammatory cells (especially monocytes/macrophages) in the aortic wall [43].

**FIGURE 8:**
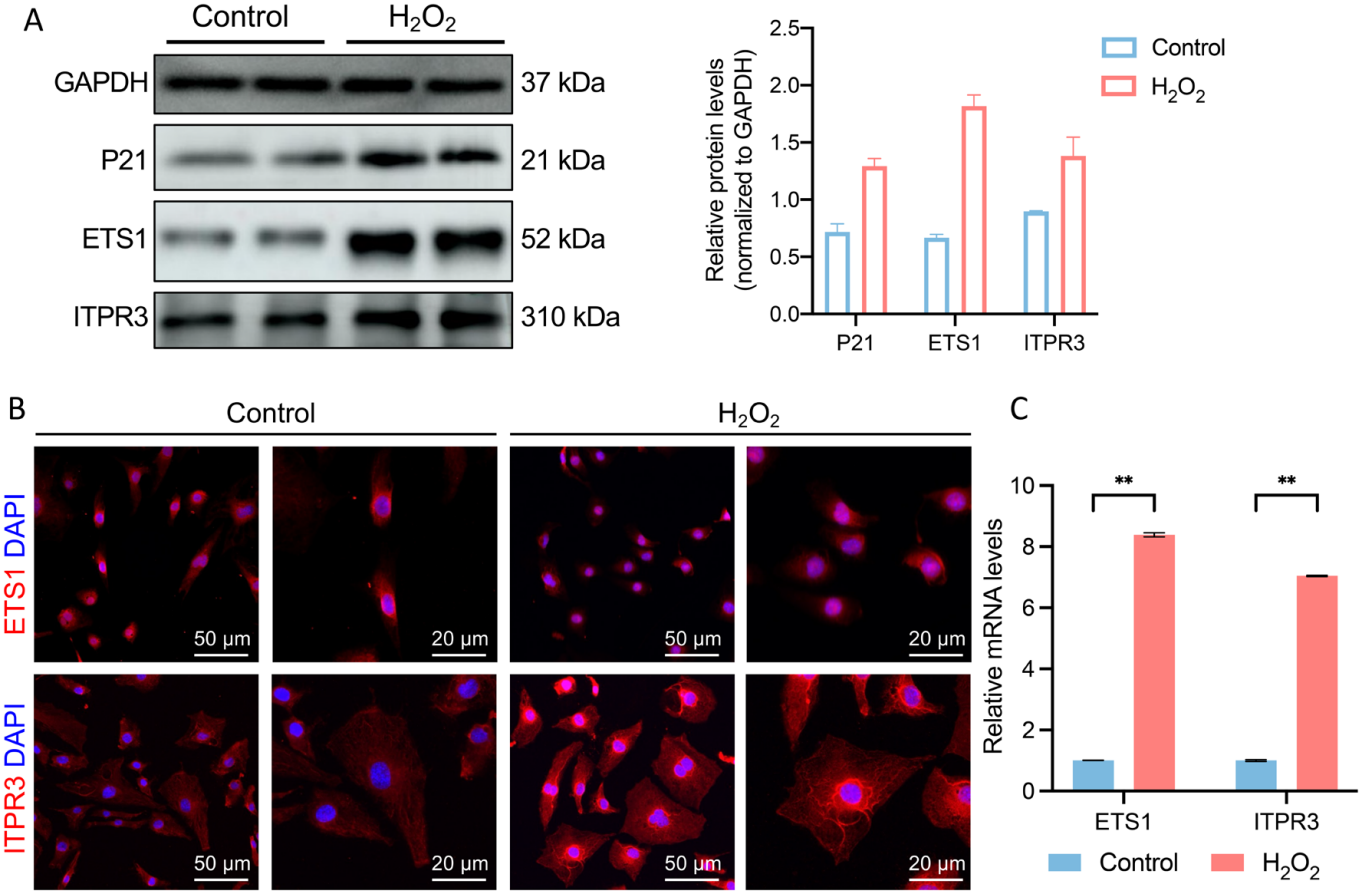
Further validation of ETS1 and ITPR3 on endothelial cells. (A) ETS1 and ITPR3 protein expression assessed by WB in HUVEC. (B) IF of ETS1 and ITPR3 in senescence and control cells. (C) Relative ETS1 and ITPR3 mRNA expression by RT‐ qPCR in HUVEC. (A) through (C) HUVEC treated with H_2_O_2_ to stimulate senescence. **p < 0.01.

Our single‐cell data analysis indicated that ETS1 was predominantly expressed in endothelial cells. To explore this further, we assessed the expression levels of ETS1 and ITPR3 in endothelial cells. We found that H_2_O_2_ stimulation induced senescence in HUVEC, resulting in significantly increased expression of senescence‐associated protein P21. Additionally, the protein levels of ETS1 and ITPR3 were significantly higher in H_2_O_2_ group (Figure 8A). Consistent changes were observed at the RNA level (Figure 8C), aligning with the aforementioned bioinformatics analysis results. Immunofluorescence detection of ETS1 and ITPR3 protein expression in cells revealed the same results (Figure 8B). It is further emphasised that ETS1 and ITPR3 may affect AAA by influencing endothelial senescence.

## 4 Discussion

Abdominal aortic aneurysm is a prevalent high‐risk disease that significantly affects quality of life in the older population. Despite advancements in surgical procedures for AAA treatment, its postoperative morbidity and mortality rates remain significant. Therefore, identification of novel diagnostic biomarkers and therapeutic targets is imperative. Several studies have unequivocally established that ageing is a significant and prominent risk factor for AAA [44]. Furthermore, compelling evidence has demonstrated the association between descending thoracic aortic plaques and accelerated brain ageing [45]. However, diagnostic biomarkers specifically associated with cellular senescence in AAA remain unclear. This study systematically screened and characterised potential cellular senescence‐related diagnostic biomarkers associated with AAA using advanced bioinformatics techniques and machine learning methods in conjunction with molecular biology experiments. Furthermore, the intricate molecular features and pathways underlying cellular senescence in AAA were revealed by utilising computational algorithms and analysing large‐scale genomic and transcriptomic datasets.

In our investigation, we revealed two genes, ETS1 and ITPR3, which are intricately linked to cellular senescence, were significantly dysregulated in individuals with AAA. We first intersected the differentially expressed genes from the AAA dataset with senescence‐related genes. Subsequently, we combined SVM‐RFE, LASSO and RF to identify potential risk genes, each algorithm offering unique analytical properties. In the analysis of gene expression data, LASSO regression can help us identify genes that are significantly associated with diseases or phenotypes. By iteratively refining the feature set, SVM‐RFE enabled us to identify the most informative genes based on the grouping variables (control and AAA). This approach ultimately allowed us to determine the optimal set of genes for distinguishing between the different conditions. As an ensemble learning method, RF constructs multiple decision trees and aggregates their predictions to achieve higher accuracy and stability. In gene selection, analysing the importance scores assigned to each gene by the RF model enables us to identify those genes with the most significant impact on AAA. This approach ensures that we can reliably pinpoint key genetic factors influencing the disease.

However, the current machine learning methods used are based on existing evidence, and they do not delve deeper into the potential biological roles and new signal transduction pathways of a gene. In other words, machine learning is evidence‐guided research, not discovery‐guided research. Therefore, it will limit the scope of gene screening research and the discovery of new molecular mechanisms. Additionally, we validated the identified hub genes in independent datasets, ROC curve analysis using the GSE183464 validation dataset confirmed that ETS1 and ITPR3 are capable of accurately diagnosing AAA. AAA can be classified into two categories based on diameter: small AAA (< 55 mm) and large AAA (≥ 55 mm). The risk of AAA rupture increases with diameter [5]. To investigate the association of ETS1 and ITPR3 with AAA expansion, we combined the GSE57691 and GSE98278 datasets as a second validation set. These datasets included critical clinical data, such as AAA size, thereby highlighting the potential of ETS1 and ITPR3 in assessing AAA progression. Furthermore, ETS1 and ITPR3 were validated through single‐cell analysis and molecular biology experiments, with ETS1 demonstrating notable efficacy. Bioinformatics, machine learning and molecular biology complement each other, demonstrating the feasibility of senescence‐related genes as novel biomarkers for AAA.

ETS1 is a member of a highly conserved family of ETS genes. ETS1 is known to regulate various genes involved in extracellular matrix remodelling, a process crucial in the progression of AAA. Specifically, ETS1 has been implicated in the regulation of MMP1, MMP3, MMP9, uPA, VEGF and VEGF receptor gene expressions [46]. ETS1 has been implicated in vascular remodelling and the recruitment of inflammatory cells, processes essential to the aneurysmal pathology, as demonstrated in animal models where ETS1 knockout reduces angiotensin II‐induced vascular changes. Additionally, ETS1 has been implicated in vascular remodelling and the recruitment of inflammatory cells, processes essential to aneurysmal pathology, as demonstrated in animal models where ETS1 knockout reduces angiotensin II–induced vascular changes [47]. The role of ETS1 extends beyond matrix regulation to the modulation of immune responses, which are critical in AAA development, as it regulates the activity of interleukin‐5 (IL‐5), contributing to the recruitment of T cells [48]. The association of ETS1 with autoimmune and inflammatory diseases [49] underscores its importance in mediating vascular inflammation, another key contributor to AAA. As an angiogenic transcription factor, ETS1 has recently been identified as a biomarker for the diagnosis of Alzheimer's disease (AD) [50]. In the cortical microvessels of AD patients, enhanced vascular immune reactivity and leakage have been observed [51]. ETS1 expression is closely associated with the expression of tumour necrosis factor‐α (TNF‐α), a key pro‐inflammatory cytokine released by reactive microglia. Our study also found that ETS1 and other genes were significantly enriched in the TNF signalling pathway. scRNA‐Seq combined with chromatin immunoprecipitation targeting ETS1 highlights its role in the inducing cell–cell crosstalk, which is crucial for cardiac development at early human embryonic stages [52]. In addition, ETS1 regulates healthy ageing by modulating ribosomal activity, leading to energy conservation and promoting healthy ageing in certain long‐lived individuals [53]. However, the role of ETS1 in cellular senescence and AAA has not been extensively investigated. Our study revealed distinct upregulation of ETS1 expression in patients with AAA. Furthermore, in patients with large AAA and ruptured AAA compared with those with small AAA, we observed an increase in ETS1 expression. This cellular senescence‐related gene, thus, emerges as a promising candidate biomarker for AAA, as validated through the validation of bioinformatics and experiments.

The inositol 1,4,5‐trisphosphate receptor (ITPR3, also known as IP3R3) is crucial for regulating intracellular calcium (Ca^2+^) homeostasis [54], a process that has been increasingly recognised as integral to cellular senescence and AAA. ITPR3 facilitates the release of Ca^2+^ from the endoplasmic reticulum, affecting a wide range of biological processes, including signal transduction, apoptosis and cytoskeleton remodelling [55]. Interestingly, ITPR3 has been shown to promote replicative senescence in fibroblasts [56], which could extend to its role in endothelial cell senescence. ITPR3 is part of the calcium release mechanism that mediates the synthesis of nitric oxide (NO) in endothelial cells, a molecule essential for maintaining vascular tone and preventing pathological dilation [57]. Multiomics investigations conducted by He et al. demonstrated that pulsed shear force enhanced ITPR3, fostering the activation of calcium ion‐dependent endothelial NO synthase and preserving endothelial cell homeostasis [58]. Moreover, a case–control study provided evidence that rs2229634, a single nucleotide polymorphism within ITPR3, was correlated with an increased risk of coronary artery aneurysm development in children [59], further supporting the hypothesis that ITPR3 may play a broader role in vascular disease beyond AAA. In our study, we observed an increase in ITPR3 expression in AAA compared with control. Additionally, this increase can also be found in the development of AAA. The elevated expression of ITPR3 in AAA may reflect disruptions in calcium homeostasis, potentially serving as a sensitive indicator of cellular stress and dysfunction in the vascular ageing process. In combination with the ELISA results, cellular senescence‐related gene ITPR3 emerges as a plausible biomarker for AAA.

Additionally, we constructed TF‐gene and gene–miRNA interaction networks to investigate the regulatory factors influencing the expression of the two diagnostic genes at both the transcriptional and posttranscriptional levels. Our analysis identified several key transcription factors, including E2F1, GATA2, HINFP and POU2F2 that play critical roles in modulating the expression of the diagnostic genes. E2F1 is a transcription factor that regulates apoptosis and the cell cycle. It suppresses cardiac neovascularisation by downregulating the expression of VEGF and PIGF [60]. Furthermore, deregulated E2F1 expression has divergent effects on VSMCs and endothelial cells, resulting in endothelial recovery and inhibition of neointimal growth [61]. The endothelial transcription factor GATA2 is downregulated in the failing myocardium of humans. GATA2 knockout causes endothelial cells to release two long noncoding RNAs (AK037972 and AK038629), which may trigger heart failure by affecting the stress reactivity of cardiomyocytes [62]. CEBPB/POU2F2 regulates the expression of endothelin 1 in VSMCs of prehypertensive spontaneously hypertensive rats [63]. miRNAs are involved in the pathological process of AAA. For instance, miR‐21 has been differentially expressed in AAA tissues in multiple studies, whereas miR‐155 and miR‐29b have been differentially expressed in both tissue‐ and blood‐based studies on AAA [64]. Additionally, one study revealed the clinical value of serum miR‐1‐3p as a potential circulating biomarker for AAA [65]. Several other miRNAs, such as miR‐17‐3p, miR‐34a and miR‐22, are involved in the pathophysiology of cellular senescence [66]. Based on the coexpression network of diagnostic genes and target miRNAs constructed in our study, hsa‐mir‐34a‐5p, hsa‐mir‐182‐5p, hsa‐mir‐129‐2‐3p, hsa‐mir‐1‐3p, hsa‐mir‐214‐3p, hsa‐mir‐374a‐5p, hsa‐let‐7d‐5p and hsa‐mir‐124‐3p were identified as common target miRNAs of ETS1 and ITPR3, suggesting their potential involvement in AAA pathology.

The identification of specific cellular SRGs like ETS1 and ITPR3 offers potential for developing biomarkers to improve early detection and risk stratification in AAA. Elevated serum levels of ETS1 and ITPR3 could be used in routine screening for high‐risk individuals, such as the elderly or those with hypertension, smoking or a family history of AAA. Beyond early detection, these biomarkers could play a crucial role in risk stratification, identifying patients at higher risk for rapid AAA progression or rupture. By combining serum marker levels with imaging studies, clinicians could refine risk models and determine the optimal timing for surgical intervention (Figure S7). These biomarkers could also be valuable for monitoring disease progression in patients undergoing conservative management, where changes in biomarker levels might indicate the need for more aggressive treatment strategies. Despite their promising, challenges remain in standardisation assays and establishing precise cut‐off levels for clinical application. Larger studies are needed to validate these biomarkers across diverse populations, considering factors such as ethnicity, age and comorbidities. Future studies should also explore combining them with machine learning models to further refine risk prediction for AAA progression. Ultimately, incorporating ETS1 and ITPR3 into diagnostic tools could enhance the accuracy and utility of AAA screening and monitoring.

Still, there are potential limitations and biases. While we employed well‐established datasets and our results are promising, they may not fully represent the diversity of patient populations, including factors such as ethnicity, age and comorbidities. This could limit the generalisability of our findings to broader patient groups. Another consideration is the small sample size, which, although sufficient for initial validation, may limit the statistical power and robustness of our conclusions. Further, larger scale studies with diverse patient cohorts will be instrumental in establishing the robustness and generalisability of these findings. Additionally, mechanistic studies are required to elucidate the precise role of ITPR3 in AAA and explore therapeutic strategies aimed at restoring calcium homeostasis to mitigate AAA progression in an ageing population. Although our study establishes an association between the cellular senescence‐related ETS1, ITPR3 and AAA, further investigation in vitro is imperative to uncover the specific molecular mechanisms.

## 5 Conclusion

The present study identifies two SRGs (ETS1 and ITPR3) as potential therapeutic targets in AAA, offering a novel perspective on the role of senescence in disease progression. Elevated levels of ETS1 and ITPR3 in serum could serve as noninvasive indicators for assessing AAA risk, particularly in instances where imaging may not detect early or subtle anatomical changes. Both biomarkers could complement imaging techniques, improving early diagnosis and risk stratification. Future research should focus on validating these findings in larger and more diverse patient populations to enhance the generalisability of these biomarkers.

## Author Contributions

**Shuli Zhang:** writing – original draft (equal). **Jiayin Li:** methodology (equal). **Ruichen Wang:** software (equal). **Xiaojie Zhao:** resources (equal). **Zhu Mei:** validation (equal). **Xiaozeng Wang:** writing – review and editing (equal).

## Ethics Statement

Serum of AAA was collected from patients diagnosed with AAA, while control serum samples were obtained from the patients of arrhythmia (the diameter of the abdominal aorta is not dilated and the abdominal aortic wall is structured).

## Consent

All samples were collected following proper ethical guidelines and with the informed consent of the patients.

## Conflicts of Interest

The authors declare no conflicts of interest.

## Supporting information


**Figure S1.** Boxplot and PCA of GEO datasets (GSE57691, GSE183464 and GSE 98278).
**Figure S2.** Boxplot and PCA of merged datasets of GSE183464 and GSE 98278.
**Figure S3.** Enrichment analyses using gene set enrichment analysis (GSEA).
**Figure S4.** The stability and predictive capabilities of the model verify by 10‐fold cross‐validation or independent validation sets.
**Figure S5.** The TF‐gene, miRNA‐gene and gene–gene interaction networks.
**Figure S6.** Functional enrichment analysis of endothelial and NK cells.
**Figure S7.** The workflow chart of inclusion of EST1 serological test indicators in clinical tests.

**Table S1.** Characteristics of the dataset.

**Table S2.** Nineteen differentially expressed senescence‐related genes.

**Table S3.** Senescence‐related genes from CellAge.

**Table S4.** Two hundred and twenty‐nine significantly up‐ and 200 downregulated genes in GSE57691.

**Table S5.** GO and KEGG enrichment of endothelials and NK cells.

## Data Availability

The original contributions presented in this study are included in the article/Supporting Information, further inquiries can be directed to the corresponding authors.
