# Supplementary material for: Cellular Senescence Genes as Cutting‐Edge Signatures for Abdominal Aortic Aneurysm Diagnosis: Potential for Innovative Therapeutic Interventions
Source: J Cell Mol Med. 2025 Jan 17;29(2):e70323. doi: 10.1111/jcmm.70323 (PMC11740988; doi:10.1111/jcmm.70323)
Supplement: Supplementary file 1 — Figure S1. Boxplot and PCA of GEO datasets (GSE57691, GSE183464 and GSE 98278). Figure S2. Boxplot and PCA of merged datasets of GSE183464 and GSE 98278. Figure S3. Enrichment analyses using gene set enrichment analysis (GSEA). Figure S4. The stability and predictive capabilities of the model verify by 10‐fold cross‐validation or independent validation sets. Figure S5. The TF‐gene, miRNA‐gene and gene–gene interaction networks. Figure S6. Functional enrichment analysis of endothelial and NK cells. Figure S7. The workflow chart of inclusion of EST1 serological test indicators in clinical tests. [file JCMM-29-e70323-s006.pdf]

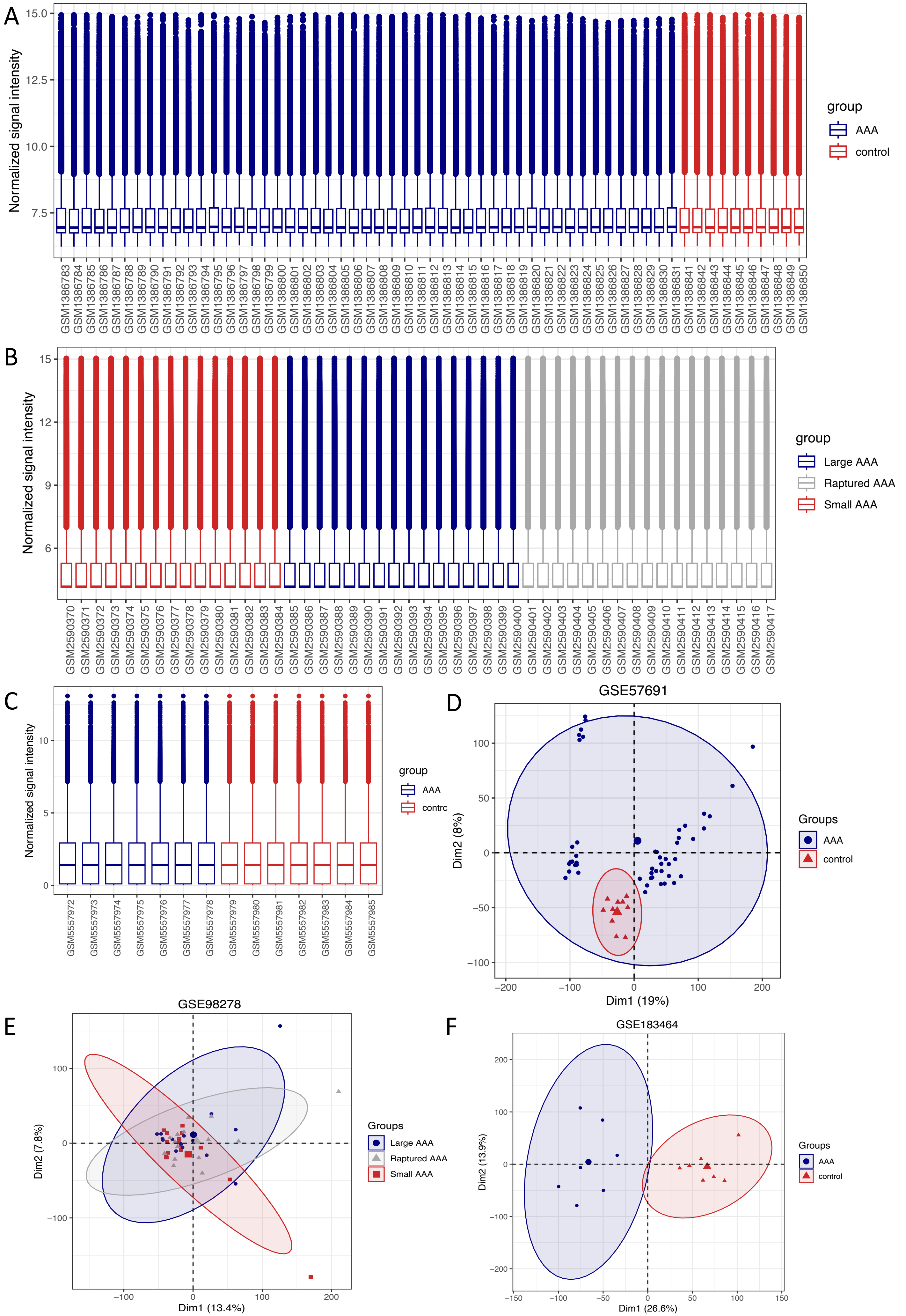

A
Normalized signal intensity
15.0
12.5
10.0
7.5
group
AAA
control
GSM1386783
GSM1386784
GSM1386785
GSM1386786
GSM1386787
GSM1386788
GSM1386789
GSM1386790
GSM1386791
GSM1386792
GSM1386793
GSM1386794
GSM1386795
GSM1386796
GSM1386797
GSM1386798
GSM1386799
GSM1386800
GSM1386801
GSM1386802
GSM1386803
GSM1386804
GSM1386805
GSM1386806
GSM1386807
GSM1386808
GSM1386809
GSM1386810
GSM1386811
GSM1386812
GSM1386813
GSM1386814
GSM1386815
GSM1386816
GSM1386817
GSM1386818
GSM1386819
GSM1386820
GSM1386821
GSM1386822
GSM1386823
GSM1386824
GSM1386825
GSM1386826
GSM1386827
GSM1386828
GSM1386829
GSM1386830
GSM1386831
GSM1386841
GSM1386842
GSM1386843
GSM1386844
GSM1386845
GSM1386846
GSM1386847
GSM1386848
GSM1386849
GSM1386850
B
Normalized signal intensity
15
12
9
6
group
Large AAA
Raptured AAA
Small AAA
GSM2590370
GSM2590371
GSM2590372
GSM2590373
GSM2590374
GSM2590375
GSM2590376
GSM2590377
GSM2590378
GSM2590379
GSM2590380
GSM2590381
GSM2590382
GSM2590383
GSM2590384
GSM2590385
GSM2590386
GSM2590387
GSM2590388
GSM2590389
GSM2590390
GSM2590391
GSM2590392
GSM2590393
GSM2590394
GSM2590395
GSM2590396
GSM2590397
GSM2590398
GSM2590399
GSM2590400
GSM2590401
GSM2590402
GSM2590403
GSM2590404
GSM2590405
GSM2590406
GSM2590407
GSM2590408
GSM2590409
GSM2590410
GSM2590411
GSM2590412
GSM2590413
GSM2590414
GSM2590415
GSM2590416
GSM2590417
C
Normalized signal intensity
10
5
0
group
AAA
contrc
GSM5557972
GSM5557973
GSM5557974
GSM5557975
GSM5557976
GSM5557977
GSM5557978
GSM5557979
GSM5557980
GSM5557981
GSM5557982
GSM5557983
GSM5557984
GSM5557985
D
GSE57691
Dim2 (8%)
100
50
0
−50
−100
−200
−100
0
100
200
Dim1 (19%)
Groups
AAA
control
E
GSE98278
Dim2 (7.8%)
100
0
−100
−100
0
100
200
Dim1 (13.4%)
Groups
Large AAA
Raptured AAA
Small AAA
F
GSE183464
Dim2 (13.9%)
200
100
0
−100
−200
−150
−100
−50
0
50
100
150
Dim1 (26.6%)
Groups
AAA
control

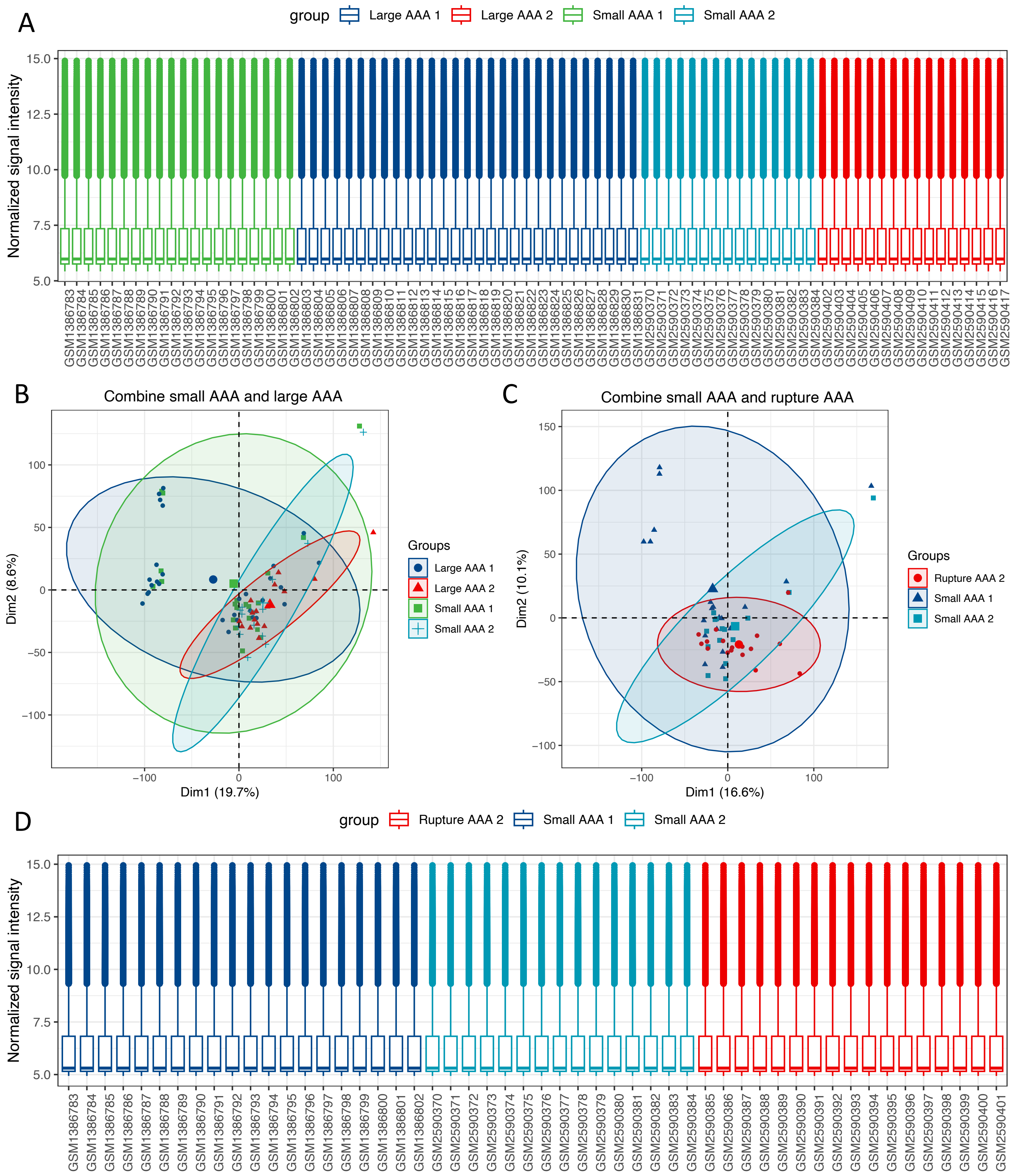
A
group
Large AAA 1
Large AAA 2
Small AAA 1
Small AAA 2
Normalized signal intensity
15.0
12.5
10.0
7.5
5.0
GSM1386783
GSM1386784
GSM1386785
GSM1386786
GSM1386787
GSM1386788
GSM1386789
GSM1386790
GSM1386791
GSM1386792
GSM1386793
GSM1386794
GSM1386795
GSM1386796
GSM1386797
GSM1386798
GSM1386799
GSM1386800
GSM1386801
GSM1386802
GSM1386803
GSM1386804
GSM1386805
GSM1386806
GSM1386807
GSM1386808
GSM1386809
GSM1386810
GSM1386811
GSM1386812
GSM1386813
GSM1386814
GSM1386815
GSM1386816
GSM1386817
GSM1386818
GSM1386819
GSM1386820
GSM1386821
GSM1386822
GSM1386823
GSM1386824
GSM1386825
GSM1386826
GSM1386827
GSM1386828
GSM1386829
GSM1386830
GSM1386831
GSM2590370
GSM2590371
GSM2590372
GSM2590373
GSM2590374
GSM2590375
GSM2590376
GSM2590377
GSM2590378
GSM2590379
GSM2590380
GSM2590381
GSM2590382
GSM2590383
GSM2590384
GSM2590402
GSM2590403
GSM2590404
GSM2590405
GSM2590406
GSM2590407
GSM2590408
GSM2590409
GSM2590410
GSM2590411
GSM2590412
GSM2590413
GSM2590414
GSM2590415
GSM2590416
GSM2590417
B
Combine small AAA and large AAA
Dim2 (8.6%)
Dim1 (19.7%)
100
50
0
−50
−100
−100
0
100
Groups
Large AAA 1
Large AAA 2
Small AAA 1
Small AAA 2
C
Combine small AAA and rupture AAA
Dim2 (10.1%)
Dim1 (16.6%)
150
100
50
0
−50
−100
−100
0
100
Groups
Rupture AAA 2
Small AAA 1
Small AAA 2
D
group
Rupture AAA 2
Small AAA 1
Small AAA 2
Normalized signal intensity
15.0
12.5
10.0
7.5
5.0
GSM1386783
GSM1386784
GSM1386785
GSM1386786
GSM1386787
GSM1386788
GSM1386789
GSM1386790
GSM1386791
GSM1386792
GSM1386793
GSM1386794
GSM1386795
GSM1386796
GSM1386797
GSM1386798
GSM1386799
GSM1386800
GSM1386801
GSM1386802
GSM2590370
GSM2590371
GSM2590372
GSM2590373
GSM2590374
GSM2590375
GSM2590376
GSM2590377
GSM2590378
GSM2590379
GSM2590380
GSM2590381
GSM2590382
GSM2590383
GSM2590384
GSM2590385
GSM2590386
GSM2590387
GSM2590388
GSM2590389
GSM2590390
GSM2590391
GSM2590392
GSM2590393
GSM2590394
GSM2590395
GSM2590396
GSM2590397
GSM2590398
GSM2590399
GSM2590400
GSM2590401

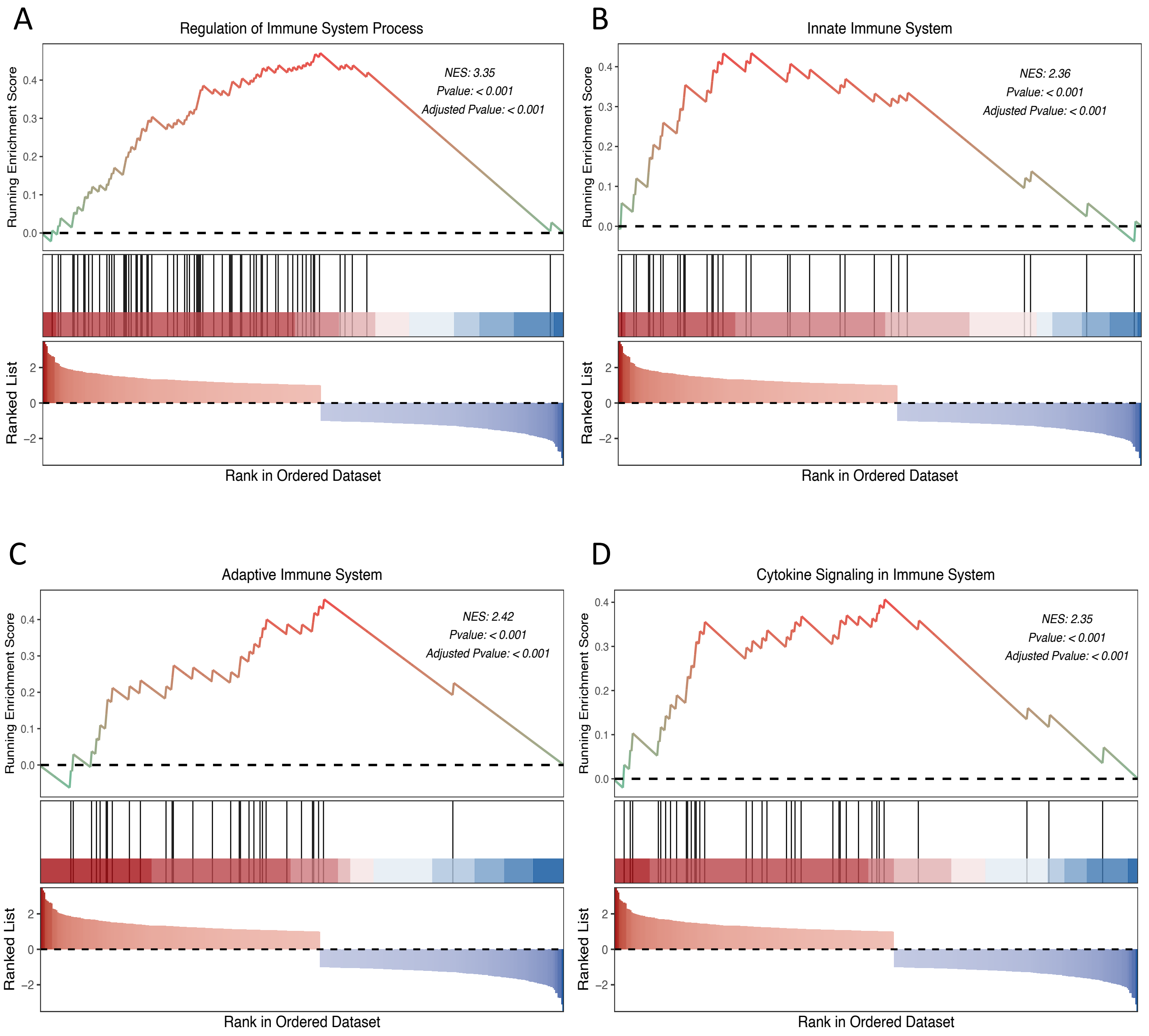
A
Regulation of Immune System Process
NES: 3.35
Pvalue: < 0.001
Adjusted Pvalue: < 0.001
Running Enrichment Score
Ranked List
Rank in Ordered Dataset
B
Innate Immune System
NES: 2.36
Pvalue: < 0.001
Adjusted Pvalue: < 0.001
Running Enrichment Score
Ranked List
Rank in Ordered Dataset
C
Adaptive Immune System
NES: 2.42
Pvalue: < 0.001
Adjusted Pvalue: < 0.001
Running Enrichment Score
Ranked List
Rank in Ordered Dataset
D
Cytokine Signaling in Immune System
NES: 2.35
Pvalue: < 0.001
Adjusted Pvalue: < 0.001
Running Enrichment Score
Ranked List
Rank in Ordered Dataset

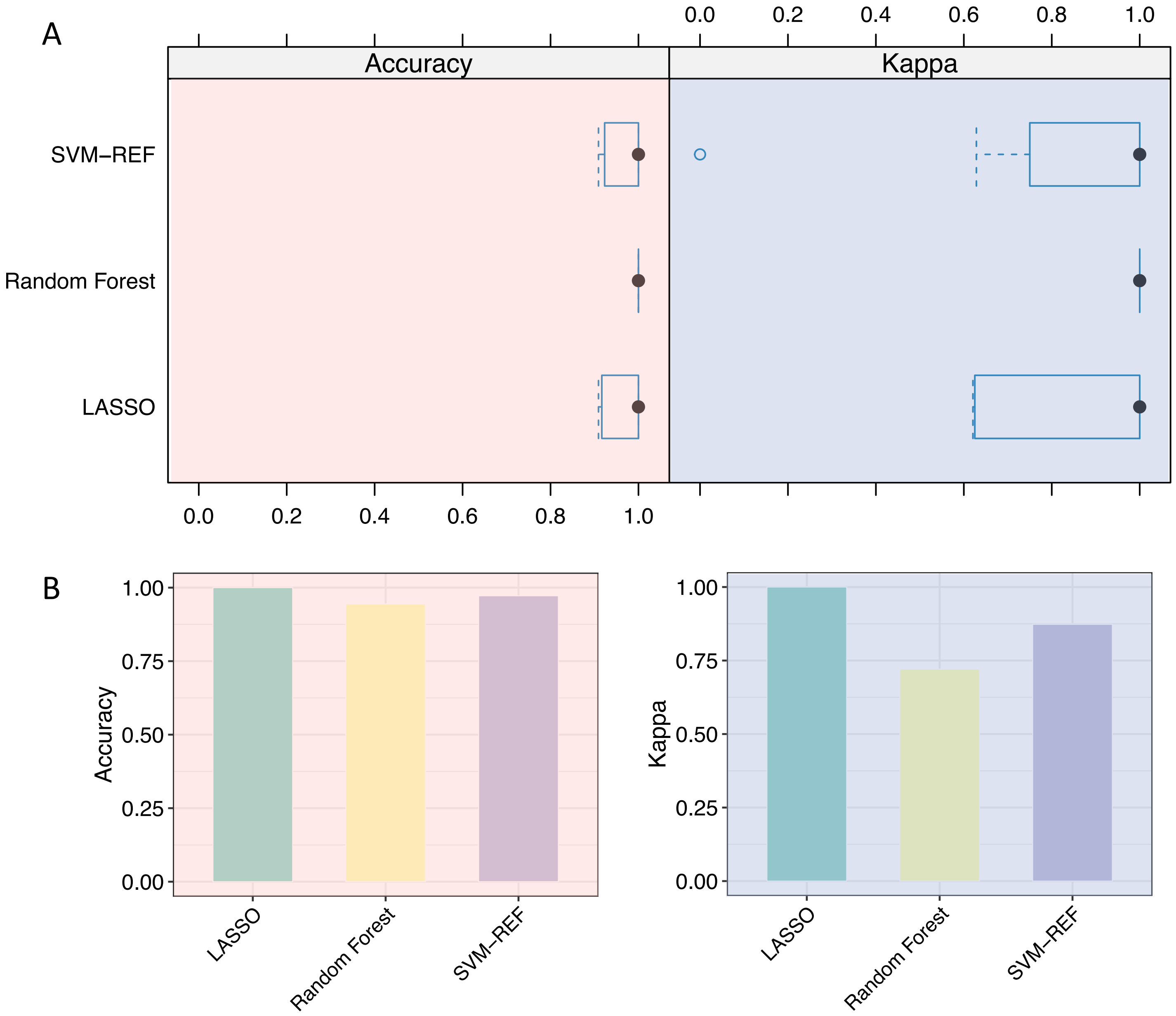

A
Accuracy
Kappa
SVM−REF
Random Forest
LASSO
0.0
0.2
0.4
0.6
0.8
1.0
B
Accuracy
Kappa
1.00
0.75
0.50
0.25
0.00
LASSO
Random Forest
SVM−REF

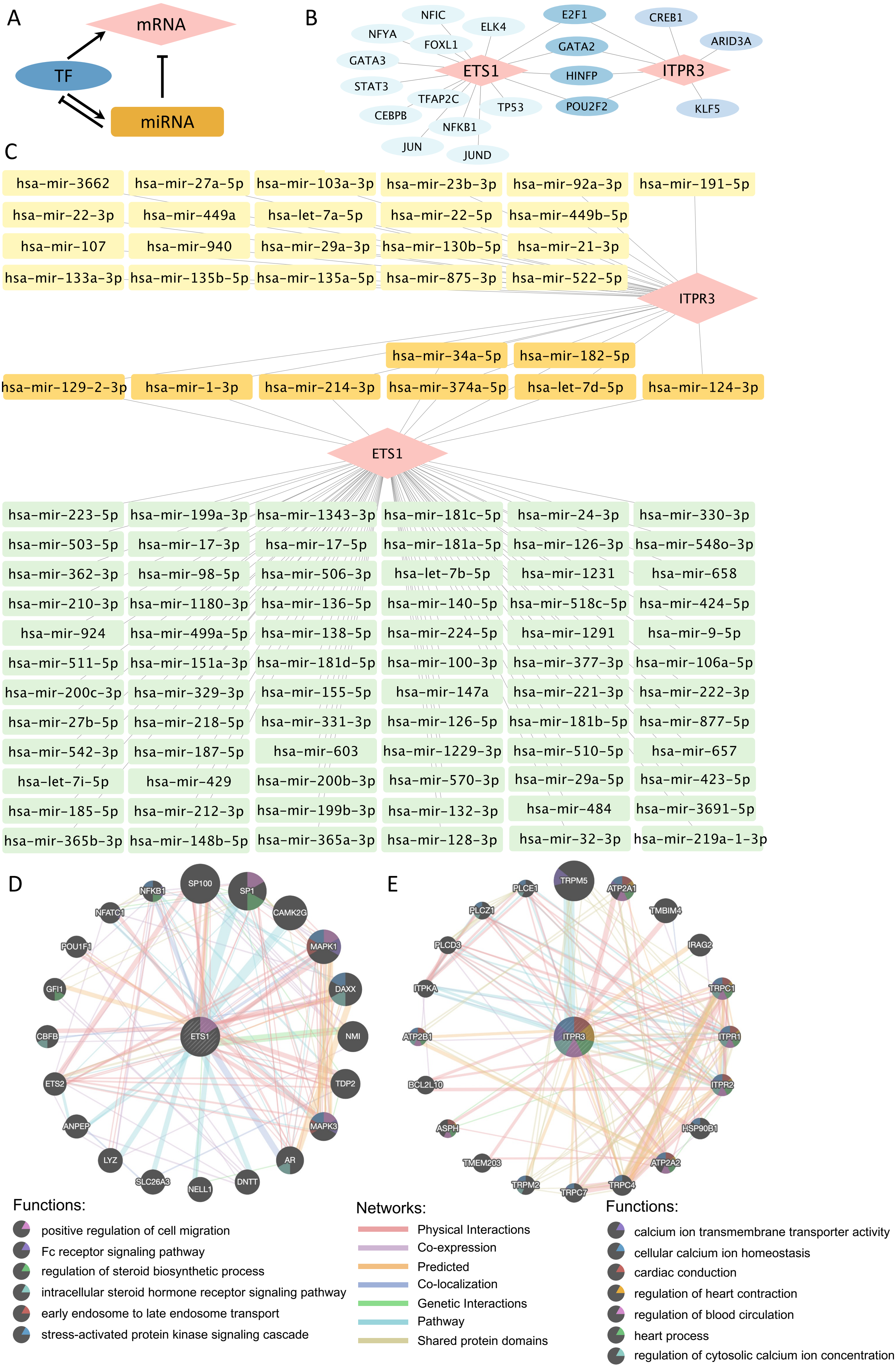

A
mRNA
TF
miRNA
B
NFIC
NFYA
ELK4
FOXL1
E2F1
CREB1
ARID3A
GATA3
GATA2
ETS1
HINFP
ITPR3
STAT3
TFAP2C
TP53
POU2F2
KLF5
CEBPB
NFKB1
JUN
JUND
C
hsa-mir-3662
hsa-mir-27a-5p
hsa-mir-103a-3p
hsa-mir-23b-3p
hsa-mir-92a-3p
hsa-mir-191-5p
hsa-mir-22-3p
hsa-mir-449a
hsa-let-7a-5p
hsa-mir-22-5p
hsa-mir-449b-5p
hsa-mir-107
hsa-mir-940
hsa-mir-29a-3p
hsa-mir-130b-5p
hsa-mir-21-3p
hsa-mir-133a-3p
hsa-mir-135b-5p
hsa-mir-135a-5p
hsa-mir-875-3p
hsa-mir-522-5p
ITPR3
hsa-mir-34a-5p
hsa-mir-182-5p
hsa-mir-129-2-3p
hsa-mir-1-3p
hsa-mir-214-3p
hsa-mir-374a-5p
hsa-let-7d-5p
hsa-mir-124-3p
ETS1
hsa-mir-223-5p
hsa-mir-199a-3p
hsa-mir-1343-3p
hsa-mir-181c-5p
hsa-mir-24-3p
hsa-mir-330-3p
hsa-mir-503-5p
hsa-mir-17-3p
hsa-mir-17-5p
hsa-mir-181a-5p
hsa-mir-126-3p
hsa-mir-548o-3p
hsa-mir-362-3p
hsa-mir-98-5p
hsa-mir-506-3p
hsa-let-7b-5p
hsa-mir-1231
hsa-mir-658
hsa-mir-210-3p
hsa-mir-1180-3p
hsa-mir-136-5p
hsa-mir-140-5p
hsa-mir-518c-5p
hsa-mir-424-5p
hsa-mir-924
hsa-mir-499a-5p
hsa-mir-138-5p
hsa-mir-224-5p
hsa-mir-1291
hsa-mir-9-5p
hsa-mir-511-5p
hsa-mir-151a-3p
hsa-mir-181d-5p
hsa-mir-100-3p
hsa-mir-377-3p
hsa-mir-106a-5p
hsa-mir-200c-3p
hsa-mir-329-3p
hsa-mir-155-5p
hsa-mir-147a
hsa-mir-221-3p
hsa-mir-222-3p
hsa-mir-27b-5p
hsa-mir-218-5p
hsa-mir-331-3p
hsa-mir-126-5p
hsa-mir-181b-5p
hsa-mir-877-5p
hsa-mir-542-3p
hsa-mir-187-5p
hsa-mir-603
hsa-mir-1229-3p
hsa-mir-510-5p
hsa-mir-657
hsa-let-7i-5p
hsa-mir-429
hsa-mir-200b-3p
hsa-mir-570-3p
hsa-mir-29a-5p
hsa-mir-423-5p
hsa-mir-185-5p
hsa-mir-212-3p
hsa-mir-199b-3p
hsa-mir-132-3p
hsa-mir-484
hsa-mir-3691-5p
hsa-mir-365b-3p
hsa-mir-148b-5p
hsa-mir-365a-3p
hsa-mir-128-3p
hsa-mir-32-3p
hsa-mir-219a-1-3p
D
SP100
SP1
NFKB1
NFATC1
CAMK2G
POU1F1
MAPK1
GFI1
DAXX
CBFB
ETS1
NMI
ETS2
TDP2
ANPEP
MAPK3
LYZ
AR
SLC26A3
NELL1
DNTT
E
TRPM5
PLCE1
ATP2A1
PLCZ1
TMBIM4
PLCD3
IRAG2
ITPKA
TRPC1
ATP2B1
ITPR3
ITPR1
BCL2L10
ITPR2
ASPH
HSP90B1
TMEM203
ATP2A2
TRPM2
TRPC7
TRPC4
Functions:
positive regulation of cell migration
Fc receptor signaling pathway
regulation of steroid biosynthetic process
intracellular steroid hormone receptor signaling pathway
early endosome to late endosome transport
stress-activated protein kinase signaling cascade
Networks:
Physical Interactions
Co-expression
Predicted
Co-localization
Genetic Interactions
Pathway
Shared protein domains
Functions:
calcium ion transmembrane transporter activity
cellular calcium ion homeostasis
cardiac conduction
regulation of heart contraction
regulation of blood circulation
heart process
regulation of cytosolic calcium ion concentration

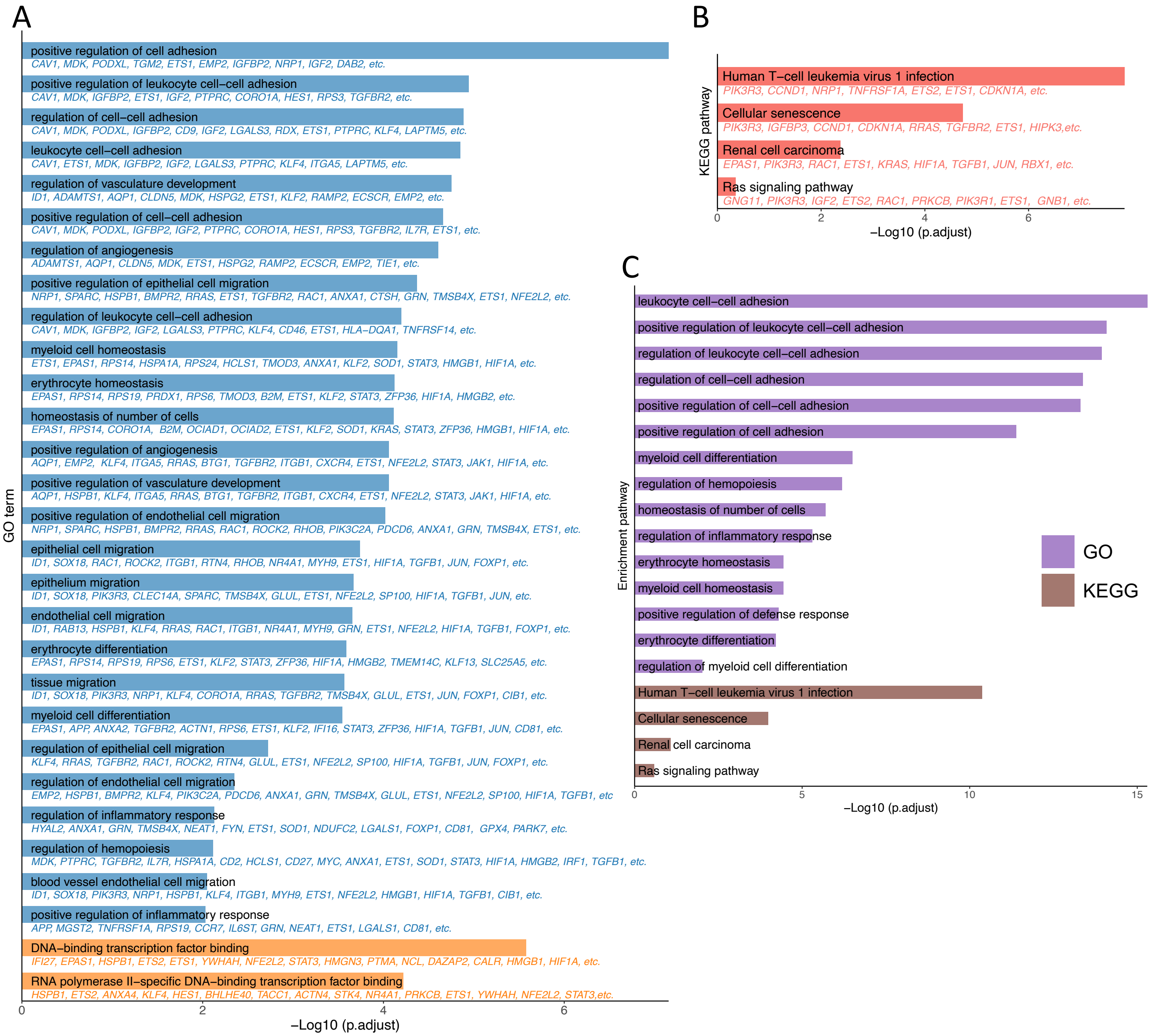
A
GO term
positive regulation of cell adhesion
CAV1, MDK, PODXL, TGM2, ETS1, EMP2, IGFBP2, NRP1, IGF2, DAB2, etc.
positive regulation of leukocyte cell−cell adhesion
CAV1, MDK, IGFBP2, ETS1, IGF2, PTPRC, CORO1A, HES1, RPS3, TGFBR2, etc.
regulation of cell−cell adhesion
CAV1, MDK, PODXL, IGFBP2, CD9, IGF2, LGALS3, RDX, ETS1, PTPRC, KLF4, LAPTM5, etc.
leukocyte cell−cell adhesion
CAV1, ETS1, MDK, IGFBP2, IGF2, LGALS3, PTPRC, KLF4, ITGA5, LAPTM5, etc.
regulation of vasculature development
ID1, ADAMTS1, AQP1, CLDN5, MDK, HSPG2, ETS1, KLF2, RAMP2, ECSCR, EMP2, etc.
positive regulation of cell−cell adhesion
CAV1, MDK, PODXL, IGFBP2, IGF2, PTPRC, CORO1A, HES1, RPS3, TGFBR2, IL7R, ETS1, etc.
regulation of angiogenesis
ADAMTS1, AQP1, CLDN5, MDK, ETS1, HSPG2, RAMP2, ECSCR, EMP2, TIE1, etc.
positive regulation of epithelial cell migration
NRP1, SPARC, HSPB1, BMPR2, RRAS, ETS1, TGFBR2, RAC1, ANXA1, CTSH, GRN, TMSB4X, ETS1, NFE2L2, etc.
regulation of leukocyte cell−cell adhesion
CAV1, MDK, IGFBP2, IGF2, LGALS3, PTPRC, KLF4, CD46, ETS1, HLA−DQA1, TNFRSF14, etc.
myeloid cell homeostasis
ETS1, EPAS1, RPS14, HSPA1A, RPS24, HCLS1, TMOD3, ANXA1, KLF2, SOD1, STAT3, HMGB1, HIF1A, etc.
erythrocyte homeostasis
EPAS1, RPS14, RPS19, PRDX1, RPS6, TMOD3, B2M, ETS1, KLF2, STAT3, ZFP36, HIF1A, HMGB2, etc.
homeostasis of number of cells
EPAS1, RPS14, CORO1A, B2M, OCIAD1, OCIAD2, ETS1, KLF2, SOD1, KRAS, STAT3, ZFP36, HMGB1, HIF1A, etc.
positive regulation of angiogenesis
AQP1, EMP2, KLF4, ITGA5, RRAS, BTG1, TGFBR2, ITGB1, CXCR4, ETS1, NFE2L2, STAT3, JAK1, HIF1A, etc.
positive regulation of vasculature development
AQP1, HSPB1, KLF4, ITGA5, RRAS, BTG1, TGFBR2, ITGB1, CXCR4, ETS1, NFE2L2, STAT3, JAK1, HIF1A, etc.
positive regulation of endothelial cell migration
NRP1, SPARC, HSPB1, BMPR2, RRAS, RAC1, ROCK2, RHOB, PIK3C2A, PDCD6, ANXA1, GRN, TMSB4X, ETS1, etc.
epithelial cell migration
ID1, SOX18, RAC1, ROCK2, ITGB1, RTN4, RHOB, NR4A1, MYH9, ETS1, HIF1A, TGFB1, JUN, FOXP1, etc.
epithelium migration
ID1, SOX18, PIK3R3, CLEC14A, SPARC, TMSB4X, GLUL, ETS1, NFE2L2, SP100, HIF1A, TGFB1, JUN, etc.
endothelial cell migration
ID1, RAB13, HSPB1, KLF4, RRAS, RAC1, ITGB1, NR4A1, MYH9, GRN, ETS1, NFE2L2, HIF1A, TGFB1, FOXP1, etc.
erythrocyte differentiation
EPAS1, RPS14, RPS19, RPS6, ETS1, KLF2, STAT3, ZFP36, HIF1A, HMGB2, TMEM14C, KLF13, SLC25A5, etc.
tissue migration
ID1, SOX18, PIK3R3, NRP1, KLF4, CORO1A, RRAS, TGFBR2, TMSB4X, GLUL, ETS1, JUN, FOXP1, CIB1, etc.
myeloid cell differentiation
EPAS1, APP, ANXA2, TGFBR2, ACTN1, RPS6, ETS1, KLF2, IFI16, STAT3, ZFP36, HIF1A, TGFB1, JUN, CD81, etc.
regulation of epithelial cell migration
KLF4, RRAS, TGFBR2, RAC1, ROCK2, RTN4, GLUL, ETS1, NFE2L2, SP100, HIF1A, TGFB1, JUN, FOXP1, etc.
regulation of endothelial cell migration
EMP2, HSPB1, BMPR2, KLF4, PIK3C2A, PDCD6, ANXA1, GRN, TMSB4X, GLUL, ETS1, NFE2L2, SP100, HIF1A, TGFB1, etc
regulation of inflammatory response
HYAL2, ANXA1, GRN, TMSB4X, NEAT1, FYN, ETS1, SOD1, NDUFC2, LGALS1, FOXP1, CD81, GPX4, PARK7, etc.
regulation of hemopoiesis
MDK, PTPRC, TGFBR2, IL7R, HSPA1A, CD2, HCLS1, CD27, MYC, ANXA1, ETS1, SOD1, STAT3, HIF1A, HMGB2, IRF1, TGFB1, etc.
blood vessel endothelial cell migration
ID1, SOX18, PIK3R3, NRP1, HSPB1, KLF4, ITGB1, MYH9, ETS1, NFE2L2, HMGB1, HIF1A, TGFB1, CIB1, etc.
positive regulation of inflammatory response
APP, MGST2, TNFRSF1A, RPS19, CCR7, IL6ST, GRN, NEAT1, ETS1, LGALS1, CD81, etc.
DNA−binding transcription factor binding
IFI27, EPAS1, HSPB1, ETS2, ETS1, YWHAH, NFE2L2, STAT3, HMGN3, PTMA, NCL, DAZAP2, CALR, HMGB1, HIF1A, etc.
RNA polymerase II−specific DNA−binding transcription factor binding
HSPB1, ETS2, ANXA4, KLF4, HES1, BHLHE40, TACC1, ACTN4, STK4, NR4A1, PRKCB, ETS1, YWHAH, NFE2L2, STAT3,etc.
0
2
4
6
−Log10 (p.adjust)
B
KEGG pathway
Human T−cell leukemia virus 1 infection
PIK3R3, CCND1, NRP1, TNFRSF1A, ETS2, ETS1, CDKN1A, etc.
Cellular senescence
PIK3R3, IGFBP3, CCND1, CDKN1A, RRAS, TGFBR2, ETS1, HIPK3,etc.
Renal cell carcinoma
EPAS1, PIK3R3, RAC1, ETS1, KRAS, HIF1A, TGFB1, JUN, RBX1, etc.
Ras signaling pathway
GNG11, PIK3R3, IGF2, ETS2, RAC1, PRKCB, PIK3R1, ETS1, GNB1, etc.
0
2
4
6
−Log10 (p.adjust)
C
Enrichment pathway
leukocyte cell−cell adhesion
positive regulation of leukocyte cell−cell adhesion
regulation of leukocyte cell−cell adhesion
regulation of cell−cell adhesion
positive regulation of cell−cell adhesion
positive regulation of cell adhesion
myeloid cell differentiation
regulation of hemopoiesis
homeostasis of number of cells
regulation of inflammatory response
erythrocyte homeostasis
myeloid cell homeostasis
positive regulation of defense response
erythrocyte differentiation
regulation of myeloid cell differentiation
Human T−cell leukemia virus 1 infection
Cellular senescence
Renal cell carcinoma
Ras signaling pathway
0
5
10
15
−Log10 (p.adjust)
GO
KEGG

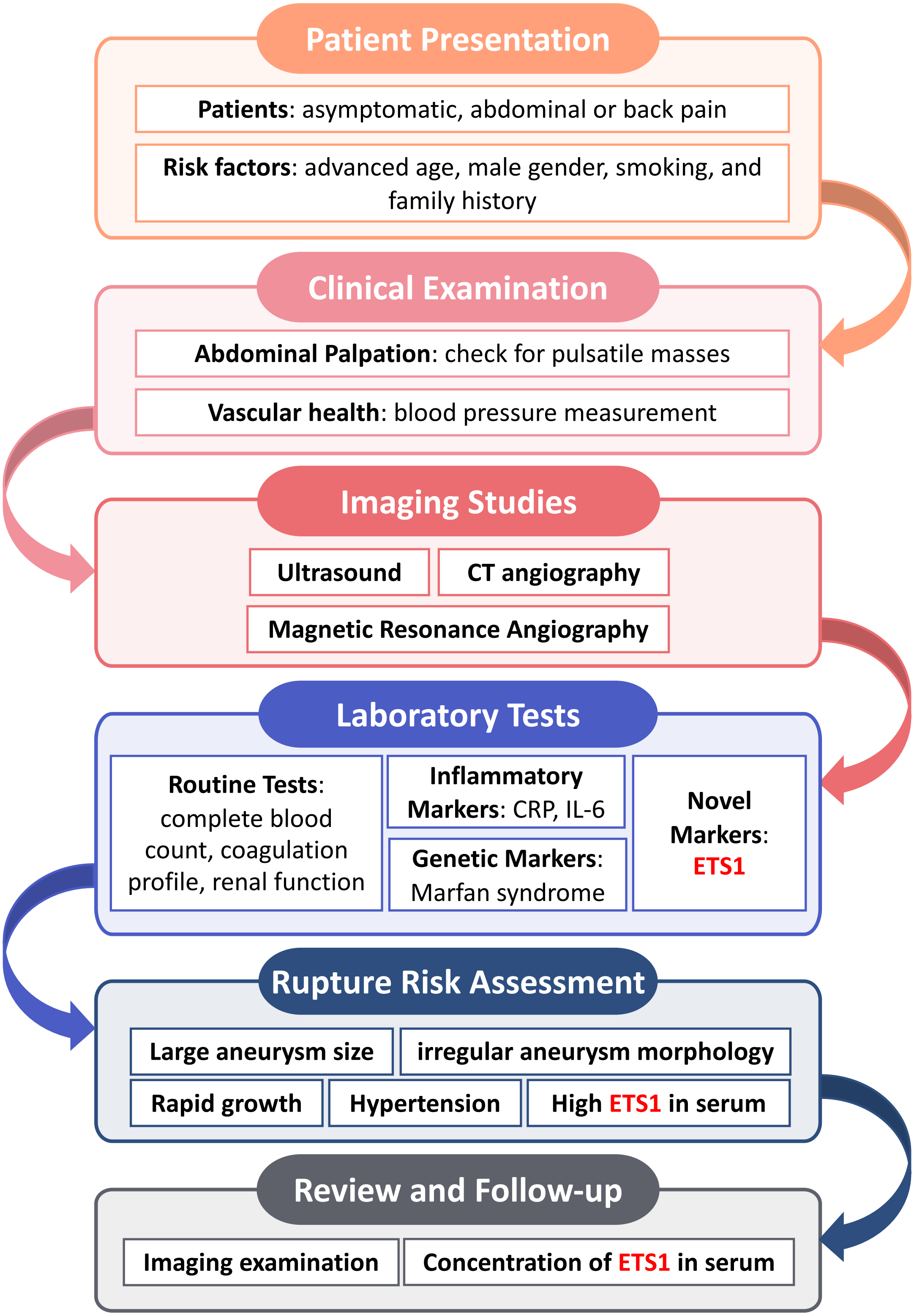
Patient Presentation
Patients: asymptomatic, abdominal or back pain
Risk factors: advanced age, male gender, smoking, and family history
Clinical Examination
Abdominal Palpation: check for pulsatile masses
Vascular health: blood pressure measurement
Imaging Studies
Ultrasound
CT angiography
Magnetic Resonance Angiography
Laboratory Tests
Routine Tests: complete blood count, coagulation profile, renal function
Inflammatory Markers: CRP, IL-6
Genetic Markers: Marfan syndrome
Novel Markers: ETS1
Rupture Risk Assessment
Large aneurysm size
irregular aneurysm morphology
Rapid growth
Hypertension
High ETS1 in serum
Review and Follow-up
Imaging examination
Concentration of ETS1 in serum
